# The uric acid-to-high-density lipoprotein cholesterol ratio, neutrophil-to-high-density lipoprotein cholesterol ratio, and lymphocyte-to-high-density lipoprotein cholesterol ratio as risk indicators for mortality in congestive heart failure: A cross-sectional analysis of NHANES 2003 to 2016

**DOI:** 10.1097/MD.0000000000049313

**Published:** 2026-06-26

**Authors:** Xinxin Wang, Yongming Liu

**Affiliations:** a The First Clinical Medical College of Lanzhou University, Lanzhou, Gansu, China; b Department of Geriatric Cardiology, The First Hospital of Lanzhou University, Gansu Provincial Clinical Research Center for Geriatric Diseases, Lanzhou, Gansu, China.

**Keywords:** congestive heart failure, lymphocyte-to-HDL ratio, mortality, neutrophil-to-HDL ratio, NHANES, serum uric acid-to-HDL cholesterol ratio

## Abstract

While individual lipid and inflammatory markers have been linked to heart failure outcomes, the prognostic value of the uric acid-to-high-density lipoprotein (HDL) cholesterol ratio (UHR), neutrophil-to-HDL cholesterol ratio (NHR), and lymphocyte-to-HDL cholesterol ratio (LHR) in predicting all-cause and cardiovascular mortality remains unclear. This study assessed the association of UHR, NHR, and LHR with mortality risk in congestive heart failure (CHF) patients. A total of 1167 CHF patients were identified from the National Health and Nutrition Examination Survey 2003 to 2016, with mortality outcomes tracked via the National Death Index through 2019. UHR, NHR, and LHR were categorized into quartiles. Associations with mortality were assessed using multivariable Cox models, Kaplan–Meier analysis, and restricted cubic splines. Prognostic performance was evaluated using receiver operating characteristic (ROC) curves, time-dependent ROC, calibration curves, and decision curve analysis. SHapley Additive exPlanations values and subgroup analyses further explored model interpretability and effect modification. During a median follow-up of 67 months, 571 (48.9%) of 1167 CHF patients died, including 235 (20.1%) cardiovascular and 336 (28.8%) non-cardiovascular deaths. After multivariable adjustment, the highest quartiles of UHR and NHR were significantly associated with increased risks of all-cause and cardiovascular mortality. For UHR, the hazard ratios were 1.03 (95% confidence interval [CI] = 1.01–1.04) for all-cause and 1.03 (95% CI = 1.01–1.05) for cardiovascular mortality. In contrast, the highest quartile of LHR was associated with a reduced risk of all-cause mortality (hazard ratio = 0.67, 95% CI = 0.50–0.90). Kaplan–Meier curves indicated worse survival in higher UHR and NHR groups (log-rank *P* < .05), while high LHR was linked to better prognosis. Restricted cubic spline analysis revealed nonlinear associations of all 3 indices with all-cause mortality, and of NHR with cardiovascular mortality. Time-dependent ROC curves showed modest discriminative ability for UHR (areas under the curve for all-cause mortality at 1, 3, 5, and 10 years: 0.581, 0.541, 0.538, and 0.563; for cardiovascular mortality: 0.610, 0.571, 0.556, and 0.586). Subgroup analysis showed a stronger UHR-mortality association in diabetics, and age-specific differences for NHR and LHR. In this study, higher levels of UHR, NHR, and LHR were associated with increased risks of all-cause and cardiovascular mortality in CHF patients. These markers, reflecting both metabolic and inflammatory status, may help identify high-risk individuals in clinical settings.

## 1. Introduction

Congestive heart failure (CHF) is a clinical syndrome resulting from systolic and/or diastolic dysfunction caused by structural or functional abnormalities of the heart due to various cardiovascular diseases. It is characterized by symptoms such as dyspnea, edema, fatigue, and elevated jugular venous pressure, representing the end stage of many cardiovascular disorders.^[1]^ With the aging global population, the prevalence of CHF continues to rise, currently affecting over 64 million people worldwide. This trend is further driven by increased survival rates after myocardial infarction (MI), the growing burden of comorbidities, and associated risk factors.^[2–4]^ Recent evidence also suggests that the incidence of CHF is increasing among individuals aged 50 years or younger.^[4]^ Therefore, how to effectively identify the prognostic risk of CHF patients has become an urgent clinical problem to be solved.

Currently, biomarkers such as B-type natriuretic peptide and N-terminal pro-B-type natriuretic peptide play essential roles in the diagnosis and severity assessment of heart failure (HF). However, their levels can be influenced by biological factors such as sex hormones; for example, testosterone may suppress natriuretic peptide secretion, leading to higher concentrations in healthy women compared to age-matched men.^[5]^ High-sensitivity troponin T can independently predict all-cause mortality, cardiovascular mortality, and hospitalization rates in HF.^[6]^ Nonetheless, in chronic or stable HF, high-sensitivity troponin T levels may remain mildly elevated or even within the normal range, limiting their prognostic utility. Moreover, its levels can be affected by various clinical conditions, reducing its specificity.^[7]^ Therefore, there is an urgent need to identify novel biomarkers or combinations thereof to improve the prediction of outcomes in patients with CHF.

Emerging evidence suggests that integrating metabolic and inflammatory markers may improve cardiovascular risk stratification, particularly in patients with CHF. The serum uric acid-to-high-density lipoprotein cholesterol ratio (UHR) has recently emerged as a novel biomarker reflecting both inflammatory and metabolic status.^[8]^ In recent decades, in addition to the well-established hemodynamic and neurohumoral mechanisms of HF, increasing evidence has highlighted the role of inflammation and metabolic disturbances in the pathogenesis and progression of CHF.^[9–12]^ Serum uric acid (SUA), a final product of purine metabolism, can contribute to endothelial dysfunction, oxidative stress, and inflammatory responses, thereby promoting HF development.^[13,14]^ Conversely, high-density lipoprotein cholesterol (HDL-C) exerts vascular protective effects, primarily through its structural protein apolipoprotein A1, which inhibits low-density lipoprotein oxidation and protects the endothelium.^[15]^ However, comorbidities affecting renal excretion and lipid metabolism limit their predictive accuracy.^[16–18]^ UHR combines the adverse implications of elevated SUA and reduced HDL-C and may offer superior prognostic value. Although previous studies have explored its association with cardiovascular outcomes in the general population, diabetic patients, and those receiving peritoneal dialysis,^[19–21]^ it has been shown that in diabetic patients, the ratio of serum UHR predicts the occurrence of cardiovascular disease more accurately than SUA and HDL-C^[8]^; however, its prognostic relevance in CHF patients remains to be clarified.

In addition to UHR, the neutrophil-to-HDL cholesterol ratio (NHR) and lymphocyte-to-HDL cholesterol ratio (LHR) have gained increasing attention. Neutrophils, as key players in innate immunity, contribute to inflammation and tissue injury during HF progression. Elevated NHR, reflecting a pro-inflammatory state relative to HDL-C’s protective role, has been associated with acute coronary syndromes and adverse cardiac outcomes.^[22]^ Conversely, LHR represents the balance between lymphocyte-mediated immune response and HDL-C-related vascular protection. Emerging evidence suggests that higher LHR may be linked to better outcomes in certain cardiovascular diseases,^[23,24]^ but its prognostic value in CHF patients remains to be fully elucidated.

We hypothesize that elevated UHR and NHR are associated with increased mortality, whereas higher LHR is associated with reduced mortality in CHF patients. This study evaluates the prognostic value of NHR, UHR, and LHR for both cardiovascular and all-cause mortality in a nationally representative cohort of CHF patients. This study aimed to utilize data from the National Health and Nutrition Examination Survey (NHANES) to evaluate the prognostic value of these ratios, providing insights for risk stratification and personalized management of CHF.

## 2. Materials and methods

### 2.1. Data sources

NHANES is a cross-sectional survey conducted by the Centers for Disease Control and Prevention in the United States (https://www.cdc.gov/nchs/nhanes/). NHANES aims to evaluate the health and nutritional status of US adults and children through interviews, physical examinations, and laboratory tests. NHANES has been approved by the NCHS Research Ethics Review Board, and all participants provided written informed consent.

### 2.2. Study populations

We downloaded data from NHANES covering the years 2003 to 2016, incorporating 7 consecutive NHANES datasets, and collected comprehensive data, including demographics, physical examinations, laboratory analyses, and questionnaires. In this retrospective cohort study, the relationship between UHR levels and individuals diagnosed with CHF was investigated. Diagnosis of CHF was confirmed by asking participants if they had a healthcare professional to inform them about their condition. CHF was based on an MCQ questionnaire, similar to published NHANES articles. Participants were asked the following question: “Did the doctor tell you that you had CHF?” Those who answered “yes” were classified as having CHF. Although the use of questionnaire feedback to define core study results may introduce some degree of uncertainty, accurate identification of HF cases is challenging given the lack of direct diagnostic evidence, such as cardiac troponin, B-type natriuretic peptide, N-terminal pro-B-type natriuretic peptide levels, or cardiac imaging tests in the NHANES database. Previous studies have demonstrated that it is feasible and accepted to use questionnaires to determine HF status in NHANES participants.^[25–27]^

Furthermore, due to the absence of echocardiographic data in NHANES, we were unable to distinguish between heart failure with reduced ejection fraction (HFrEF) and heart failure with preserved ejection fraction (HFpEF). Additional clinical information that is not collected in NHANES includes left ventricular ejection fraction, New York Heart Association functional class, and the specific clinical indications for medication use. These inherent limitations of the NHANES database should be considered when interpreting our findings. Despite these inherent data limitations, this study represents a necessary exploration of the prognostic value of these readily accessible and cost-effective biomarkers in a nationally representative cohort of patients with CHF, with the potential to inform risk assessment in settings where detailed clinical data are unavailable.

**Figure 1.**
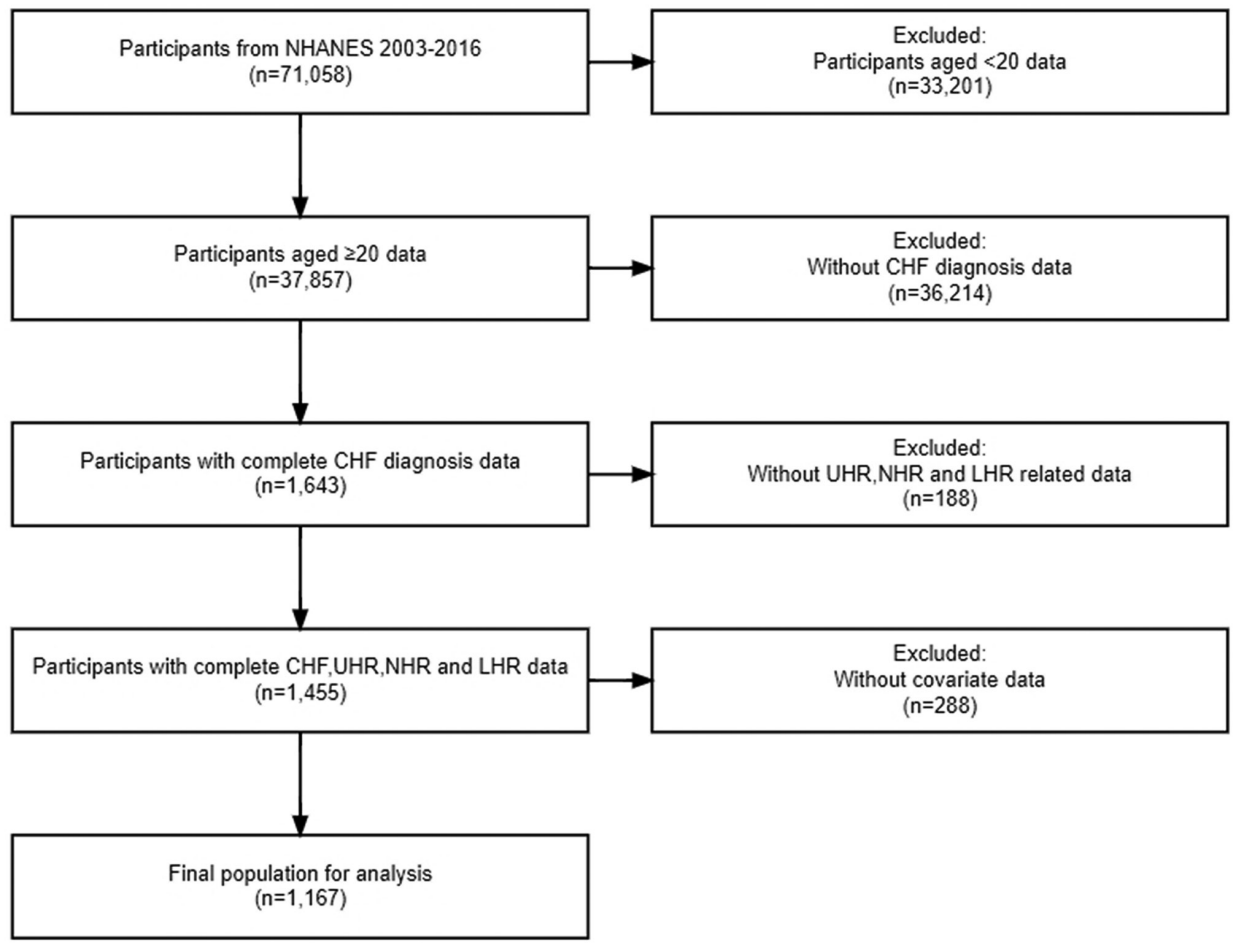
Flowchart of participant inclusion and exclusion in the current study. CHF = congestive heart failure, LHR = lymphocyte-to-high-density lipoprotein cholesterol ratio, NHANES = National Health and Nutrition Examination Survey, NHR = neutrophil-to-high-density lipoprotein cholesterol ratio, UHR = uric acid-to-high-density lipoprotein cholesterol ratio.

A total of 71,085 people participated in the NHANES survey with the following exclusion criteria: individuals under 20 years of age (n = 37,857); individuals without a diagnosis of CHF (n = 36,214); individuals with incomplete records of SUA and HDL-C (n = 188); and individuals with missing relevant covariates (n = 288). Ultimately, 1167 participants were included in the final analysis (Fig. 1).

### 2.3. Definition and calculation of UHR, LHR, and NHR

During the examination, the investigators collected blood samples from participants to measure SUA, lymphocyte count, neutrophil count, and HDL, which reflect metabolic levels and inflammatory levels. In this study, the composite indices reflecting metabolic and inflammatory status included UHR, LHR, and NHR, calculated as follows:

UHR^[28]^:


$$UHR=\frac{Serum\;\;\;uric\;\;\;acid\;\;\;\left(mg/dL\;\right)}{HDL\text{\textasciitilde{}}\text{-}C\;\left(mg/dL\;\right)}.$$

LHR^[29]^:


$$\begin{array}{c} LHR=\frac{Lymphocyte\;\;\;count\;\;\;\left(1000\;cells/\mu L\;\right)}{HDL\text{\textasciitilde{}}\text{-}C\;\;\;\left(mg/dL\;\right)}. \end{array}$$

NHR^[30]^:


$$\begin{array}{c} NHR=\frac{Neutrophil\;\;\;count\;\;\;\;\;\left(1000\;cells/\;\mu \;L\;\right)}{HDL\text{\textasciitilde{}}\text{-}C\;\;\;\left(mg/dL\;\right)}. \end{array}$$

Participants were stratified into 4 groups (Q1, Q2, Q3, and Q4) based on the quartiles of each index, with the group. This categorization was employed because established clinical cutoff values for these novel ratios are currently unavailable. Quartile-based analysis allows for the examination of potential nonlinear relationships with mortality outcomes without imposing linear assumptions, enhances the clinical interpretability of the results by comparing risk across distinct population segments.

### 2.4. Assessment of covariates

To illustrate the independent associations among UHR, LHR, NHR, and CHF, potential covariates that could influence the association among these indices and CHF were adjusted based on clinical relevance, and information on sociodemographic, lifestyle, and health status factors was collected from NHANES household interviews. Details of this analytical process are fully described in the NHANES laboratory operations manual. Sociodemographic and lifestyle-related variables included age (years), sex (male/female), race (Mexican American/other Hispanic/non-Hispanic White/non-Hispanic Black/other race), marital status (married/roll/divorced/separated/never married/living with partner), educational level (<9 years, 9–11 years, high school, some college, college graduate), and family economic status as determined by the paucity to poverty ratio, divided into 3 categories: <1.30, 1.31 to 3.50, and ≥3.50, smoking and alcohol consumption. Alcohol consumption was divided into nondrinkers (<12 drinks in 1 lifetime), ex-drinkers (more than 12 drinks in 1 lifetime and 0 drinks in the past 12 months), current drinkers (more than 12 drinks in 1 lifetime and more than 1 day in 12 months), and absence of alcohol status. Smoking was classified as nonsmoking (<100 cigarettes per lifetime), current smoking (more than 100 cigarettes per lifetime, daily or occasional smoking), former smoking (more than 100 cigarettes per lifetime, no smoking), and missing smoking status. It is important to note that the cigarette count here refers to the number of individual cigarettes, not packs. Body mass index (BMI) is measured directly by a professional at a mobile examination center and is calculated by dividing the individual’s weight (kg) by the square of the height (m; kg/m^2^).^[17]^ Clinical parameters such as white blood cell (WBC), red blood cell, hemoglobin, platelet, alanine aminotransferase, aspartate aminotransferase, blood uric acid, serum creatinine (SCr), fasting blood glucose, lymphocytes, total cholesterol (TC), HDL-C, and glycosylated hemoglobin (HbA1c) were measured in the NHANES laboratory. Missing covariate data (<5%) were present in the study population and multiple imputations using the Mice package in R language were used to improve the data. To address the missing covariate data (<5% for any variable and assumed to be missing at random), we performed multiple imputation using the mice package in R. Missing data patterns were examined with md.pattern() and visualized using the VIM package. Five complete datasets (*m* = 5) were generated using predictive mean matching for continuous variables and logistic regression for binary variables, with 50 iterations to ensure convergence and a fixed seed (seed = 1024) for reproducibility. Primary analyses were conducted on each imputed dataset, and results were pooled using Rubin’s rules to account for imputation uncertainty.

Hypertension was defined as systolic blood pressure ≥140 mm Hg/diastolic blood pressure ≥90 mm Hg and self-reported hypertension, and diabetes was defined as fasting blood glucose ≥6.1 mmol/L, HbA1c ≥6.5%, taking hypoglycemic agents, or self-reported diabetes, while data on coronary heart disease, angina pectoris, heart attack, and malignancy were based on self-reported information from participants’ questionnaires.

### 2.5. Ascertainment of mortality

The outcome measures of this study were cardiovascular death and all-cause death. Mortality status and cause of death were determined by the NDI Public Access File as of December 31, 2019, associated with NHANES. All-cause mortality was defined as death from any cause. According to the 10th Revision of the International Classification of Diseases, cardiovascular mortality was defined as deaths caused by heart disease (codes I00–I09, I11, I13, I20–I51, encompassing conditions such as rheumatic, hypertensive, and ischemic heart diseases) and cerebrovascular disease (codes I60–I69, including cerebral hemorrhage and infarction).

### 2.6. Data analysis

Statistical analyses were performed using R (version 4.4.3; R Foundation for Statistical Computing, https://www.R-project.org/) and SPSS (IBM Corp.). *P* values <.05 were considered significant. NHANES sample weights and complex survey design were applied to ensure national representativeness. Continuous data are shown as mean ± standard deviation or median (interquartile range), categorical data as counts (%). The 25th, 50th, and 75th percentiles of UHR, NHR, and LHR were calculated; UHR quartile cutoffs were 9.25, 12.81, and 17.44, with NHR and LHR cutoffs in Tables S1 and S2, Supplemental Digital Content 1. Baseline characteristics were compared across UHR quartiles using Wilcoxon rank-sum for continuous variables and chi-square or Fisher exact tests for categorical variables.

Participants were divided into UHR quartiles (Q1–Q4). Mortality rates were compared across quartiles, and multivariate Cox regression models assessed their independent predictive value. Model 1 was unadjusted; Model 2 adjusted for age, sex, race; Model 3 further adjusted for BMI, education, income, malignancy, smoking, drinking status, SCr, WBC, ALB, TC, hypertension, diabetes, and coronary heart disease.

Survival differences were examined by Kaplan–Meier curves and log-rank tests. Restricted cubic spline (RCS) Cox models explored nonlinear associations.^[31]^ Subgroup and interaction analyses were conducted for potential effect modifiers.

Time-dependent receiver operating characteristic (ROC) curves (using the “time ROC” package) evaluated UHR’s predictive accuracy for survival, adjusting for multiple covariates.^[32]^ ROC analysis compared predictive values of UHR, NHR and LHR. Decision curve analysis (DCA) and calibration curves were generated via the “dcurves” and “rms” packages. SHapley Additive exPlanations (SHAP) values were calculated and visualized using “kernelshap” and “shapviz” to assess feature contributions.

## 3. Results

### 3.1. Baseline characteristics of the study population

This study initially screened participants according to predefined inclusion and exclusion criteria, resulting in a final sample of 1167 patients diagnosed with CHF (mean age 67.18 ± 12.1 years; 55.3% male). The detailed patient inclusion and exclusion process is illustrated in Figure 1. During the follow-up period, 571 (44%) participants died from all causes, and 235 (18%) died due to cardiovascular causes.

Table 1 presents the weighted distributions of sociodemographic and clinical characteristics across quartiles of the serum UHR. The UHR quartile cutoffs were <9.25, 9.25 to 12.81, 12.81 to 17.44, and >17.44, respectively. Significant differences were observed among quartiles for variables including sex, marital status, BMI, waist circumference, blood pressure, glucose levels, and several biochemical markers (*P* < .05). Higher UHR levels were associated with older age, male sex, obesity, and increased prevalence of comorbidities such as coronary heart disease (50%), hypertension (81%), diabetes (58%), and malignancy (25%). Participants in the highest UHR quartile also had significantly higher all-cause (51%) and cardiovascular mortality rates (22%). Elevated SCr, triglycerides, TC, fasting plasma glucose, HbA1c, SUA, blood urea nitrogen, alkaline phosphatase, and WBC counts, along with decreased HDL-C, were observed in this group (Table 1).

**Table 1:** Clinical characteristics of the study participants based on UHR index quartiles.

Characteristic	N	Overall	Q1 (<9.25)	Q2 (9.25–12.81)	Q3 (12.81–17.44)	Q4 (>17.44)	*P* value
		N = 1167	N = 269	N = 292	N = 304	N = 302	
Sex	1167						<.001
Male		646 (54%)	91 (34%)	153 (52%)	196 (64%)	206 (66%)	
Female		521 (46%)	178 (66%)	139 (48%)	108 (36%)	96 (34%)	
Age (yr)	1167	67 ± (12)	67 ± (14)	67 ± (12)	67 ± (11)	67 ± (12)	.5
Race	1167						.2
Mexican		100 (3.0%)	20 (2.7%)	27 (3.2%)	35 (4.0%)	18 (2.3%)	
American		76 (2.8%)	13 (2.7%)	28 (3.8%)	16 (1.9%)	19 (2.9%)	
Other Hispanic		710 (78%)	159 (74%)	169 (78%)	183 (81%)	199 (80%)	
Non-Hispanic		206 (9.1%)	58 (12%)	42 (6.7%)	56 (9.6%)	50 (8.6%)	
White		75 (6.6%)	19 (8.7%)	26 (8.0%)	14 (3.9%)	16 (6.0%)	
Education	1167						.8
<9th grade		188 (10%)	39 (8.8%)	52 (10%)	41 (8.3%)	56 (13%)	
9–11th grade		212 (16%)	40 (13%)	60 (18%)	59 (17%)	53 (18%)	
High school grad/GED		291 (26%)	67 (25%)	71 (23%)	80 (26%)	73 (27%)	
Some college		298 (29%)	72 (32%)	70 (30%)	76 (30%)	80 (25%)	
College graduate or above		178 (19%)	51 (21%)	39 (18%)	48 (18%)	40 (17%)	
Marital	1167						.018
Married		605 (55%)	121 (49%)	156 (56%)	151 (53%)	177 (64%)	
Widowed		276 (20%)	73 (24%)	73 (20%)	71 (20%)	59 (16%)	
Divorced		141 (11%)	28 (9.8%)	30 (8.0%)	45 (16%)	38 (11%)	
Separated		34 (2.6%)	15 (4.3%)	5 (1.1%)	7 (2.3%)	7 (2.7%)	
Single		74 (6.8%)	23 (9.6%)	16 (7.0%)	22 (6.2%)	13 (4.5%)	
Living with partner		37 (3.9%)	9 (3.2%)	12 (7.9%)	8 (2.4%)	8 (2.3%)	
PIR	1167						.3
<1.30		425 (27%)	88 (24%)	108 (25%)	121 (30%)	108 (27%)	
1.31–3.50		499 (46%)	127 (53%)	125 (45%)	118 (39%)	129 (46%)	
≥3.50		243 (28%)	54 (23%)	59 (30%)	65 (31%)	65 (26%)	
SBP (mm Hg)	1167	129 ± (20)	128 ± (19)	130 ± (19)	128 ± (20)	128 ± (22)	.8
DBP (mm Hg)	1167	70 ± (16)	69 ± (17)	71 ± (17)	69 ± (15)	70 ± (16)	.5
BMI (kg/m^2^)	1167	30 ± (7)	28 ± (6)	30 ± (7)	32 ± (7)	33 ± (6)	<.001
WAIST (cm)	1167	107 ± (17)	98 ± (15)	106 ± (16)	111 ± (17)	114 ± (15)	<.001
HbA1c (%)	1167	6.24 ± (1.27)	5.88 ± (1.09)	6.39 ± (1.37)	6.20 ± (1.11)	6.50 ± (1.41)	<.001
FBG (mmol/L)	1167	6.81 ± (2.44)	6.12 ± (1.98)	7.02 ± (2.70)	6.70 ± (2.24)	7.42 ± (2.61)	<.001
TC (mg/dL)	1167	177 ± (45)	188 ± (45)	175 ± (40)	173 ± (42)	173 ± (50)	.005
LDL (mg/dL)	1167	193 ± (256)	152 ± (181)	206 ± (235)	213 ± (261)	201 ± (323)	.079
HDL (mg/dL)	1167	50 ± (16)	68 ± (17)	51 ± (10)	44 ± (8)	35 ± (7)	<.001
TG (mg/dL)	1167	153 ± (128)	104 ± (62)	136 ± (77)	156 ± (107)	219 ± (193)	<.001
SUA (mg/dL)	1167	6.15 ± (1.71)	4.67 ± (1.11)	5.63 ± (1.09)	6.49 ± (1.12)	7.80 ± (1.67)	<.001
ALP (IU/L)	1167	74 ± (28)	74 ± (28)	74 ± (31)	74 ± (27)	74 ± (26)	>.9
ALT (IU/L)	1167	25 ± (42)	21 ± (10)	25 ± (17)	24 ± (12)	29 ± (80)	.033
AST (IU/L)	1167	26 ± (20)	26 ± (12)	25 ± (8)	26 ± (13)	28 ± (36)	>.9
ALB (g/L)	1167	4.13 ± (0.34)	4.14 ± (0.36)	4.15 ± (0.31)	4.12 ± (0.35)	4.10 ± (0.34)	.4
TBil (mmol/L)	1167	12.2 ± (5.4)	11.1 ± (4.8)	12.5 ± (5.3)	12.5 ± (5.6)	12.7 ± (5.6)	.006
LDH (IU/L)	1167	140 ± (33)	143 ± (32)	137 ± (33)	138 ± (32)	142 ± (36)	.14
P (mmol/L)	1167	1.22 ± (0.19)	1.22 ± (0.17)	1.23 ± (0.21)	1.20 ± (0.20)	1.22 ± (0.18)	.3
SCr (µmol/L)	1167	9864 ± (6124)	8689 ± (5344)	9814 ± (6163)	10,680 ± (6608)	10,276 ± (6162)	.018
BUN (mg/dL)	1167	19 ± (10)	16 ± (7)	18 ± (9)	18 ± (8)	23 ± (14)	<.001
WBC (1000 cells/µL)	1167	7.68 ± (2.53)	7.27 ± (2.19)	7.61 ± (2.10)	7.61 ± (2.83)	8.22 ± (2.82)	<.001
Lymphocyte (1000 cells/µL)	1167	26 ± (9)	27 ± (9)	26 ± (8)	26 ± (10)	25 ± (9)	.4
Monocyte (1000 cells/µL)	1167	8.53 ± (2.45)	8.79 ± (2.45)	8.53 ± (2.13)	8.27 ± (2.12)	8.52 ± (2.99)	.2
Neutrophil (1000 cells/µL)	1167	4.77 ± (1.80)	4.49 ± (1.82)	4.77 ± (1.79)	4.73 ± (1.75)	5.10 ± (1.81)	<.001
PLT (1000 cells/µL)	1167	222 ± (73)	228 ± (80)	219 ± (68)	218 ± (71)	223 ± (75)	.6
Alcohol	1167						.5
Never		455 (33%)	113 (36%)	116 (29%)	106 (33%)	120 (33%)	
Former		492 (47%)	105 (45%)	125 (51%)	133 (43%)	129 (49%)	
Current		220 (20%)	51 (19%)	51 (20%)	65 (24%)	53 (18%)	
Smoking	1167						.3
Never		456 (39%)	125 (44%)	118 (39%)	105 (37%)	108 (34%)	
Former		239 (22%)	56 (23%)	62 (25%)	63 (19%)	58 (22%)	
Current		472 (39%)	88 (33%)	112 (37%)	136 (44%)	136 (44%)	
Hypertension	1167	943 (76%)	209 (75%)	227 (68%)	249 (80%)	258 (81%)	.032
Diabetes	1167	542 (44%)	88 (28%)	133 (44%)	146 (44%)	175 (58%)	<.001
CAD	1167						<.001
Yes		446 (34%)	86 (30%)	100 (30%)	108 (30%)	152 (48%)	
No		721 (66%)	183 (70%)	192 (70%)	196 (70%)	150 (52%)	
AP	1167						.2
Yes		333 (30%)	72 (26%)	77 (28%)	85 (32%)	99 (35%)	
No		834 (70%)	197 (74%)	215 (72%)	219 (68%)	203 (65%)	
MI	1167						.3
Yes		559 (47%)	120 (41%)	129 (47%)	153 (52%)	157 (50%)	
No		608 (53%)	149 (59%)	163 (53%)	151 (48%)	145 (50%)	
Stroke	1167						.9
Yes		224 (17%)	55 (19%)	58 (16%)	54 (16%)	57 (18%)	
No		943 (83%)	214 (81%)	234 (84%)	250 (84%)	245 (82%)	
Cancer	1167						.4
Yes		250 (23%)	70 (26%)	42 (19%)	66 (23%)	72 (25%)	
No		917 (77%)	199 (74%)	250 (81%)	238 (77%)	230 (75%)	

All estimates were adjusted for the survey weights of NHANES (Q1: N = 1,389,379^2^, Q2: N = 1,386,901^2^, Q3: N = 1,384,884^2^, Q4: N = 1,386,634^2^). Baseline characteristics of participants. Continuous variables are presented as the weighted mean ± standard error. Categorical variables are presented as an unweighted number (%). All estimates were adjusted for the survey weights of NHANES.

ALT = alanine aminotransferase, AP = angina pectoris, AST = aspartate aminotransferase, BMI = body mass index, CAD = coronary artery disease, DBP = mean diastolic blood pressure, FBG = fasting blood glucose, HbA1c = glycosylated hemoglobin, HDL-C = high-density lipoprotein cholesterol, MI = myocardial infarction, PIR = poverty-to-income ratio, PLT = platelet count, SBP = mean systolic blood pressure, TBil = total bilirubin, TC = total cholesterol, TG = triglycerides, UHR = uric acid-to-high-density lipoprotein cholesterol ratio, WBC = white blood cell count, WC = waist circumference.

Similarly, higher quartiles of NHR and LHR were associated with distinct demographic and clinical features, including male sex, younger age, Mexican ethnicity, marital status, lower education, obesity, elevated glucose, decreased HDL, increased SUA and WBC counts, reduced neutrophil counts, and histories of diabetes and cardiovascular disease (*P* < .05). Baseline characteristics by quartiles of UHR, NHR, and LHR are detailed in Tables 1 and S1 and S2, Supplemental Digital Content 1.

### 3.2. Comparison of survival outcomes in different levels of UHR, NHR, and LHR groups

Univariate Cox regression analysis showed that UHR, NHR, and LHR were significantly associated with both all-cause mortality and CVD mortality in CHF patients (*P* < .05). Patients in the higher quartiles of these indices exhibited significantly increased risks compared to those in the lower quartiles (Table S3, Supplemental Digital Content 2). In addition, age, education level, marital status, poverty-to-income ratio (PIR), systolic blood pressure, BMI, SUA, lymphocytes, monocytes, HbA1c, blood urea nitrogen, diabetes, coronary heart disease, stroke, and cancer were significantly associated with all-cause and cardiovascular mortality (*P* < .05).

Three multivariate Cox regression models were constructed to examine the independent associations between the combined indices and the risks of all-cause and CVD mortality (Table 2). In the fully adjusted model (Table 2), elevated UHR and NHR levels were independently associated with higher all-cause mortality (UHR: hazard ratio [HR] = 1.42, 95% confidence interval [CI] = 1.04–1.94, *P* = .04; NHR: HR = 1.51, 95% CI = 1.12–2.04, *P* = .007) and cardiovascular mortality (UHR: HR = 2.02, 95% CI = 1.28–3.19, *P* = .002; NHR: HR = 2.10, 95% CI = 1.35–3.26, *P* < .001).

**Table 2:** Association of combined index (UHR, NHR, and LHR) with all-cause mortality and cardiovascular mortality in cardiovascular disease.

	Model 1		Model 2		Model 3	
	HR (95% CI)	*P* value	HR (95% CI)	*P* value	HR (95% CI)	*P* value
UHR: all-cause mortality						
Continuous	1.03 (1.01–1.04)	.002	1.03 (1.01–1.05)	<.001	1.03 (1.01–1.04)	.002
Quartile						
Q1	Ref		Ref		Ref	
Q2	0.89 (0.65–1.22)	.483	0.95 (0.71–1.27)	.732	0.99 (0.76–1.29)	.927
Q3	0.98 (0.72–1.34)	.905	1.05 (0.77–1.43)	.739	1.05 (0.80–1.39)	.705
Q4	1.28 (0.95–1.74)	.107	1.43 (1.04–1.97)	.026	1.42 (1.04–1.94)	.029
*P* for trend		.106		.031		.041
UHR: cardiovascular mortality						
Continuous	1.03 (1.01–1.05)	.001	1.04 (1.02–1.06)	<.001	1.04 (1.01–1.06)	.001
Quartile						
Q1	Ref		Ref		Ref	
Q2	1.07 (0.69–1.67)	.748	1.18 (0.81–1.72)	.388	1.27 (0.86–1.88)	.227
Q3	1.21 (0.73–2.01)	.465	1.36 (0.82–2.25)	.228	1.43 (0.86–2.38)	.166
Q4	1.61 (1.09–2.38)	.018	1.88 (1.23–2.87)	.003	2.02 (1.28–3.19)	.002
*P* for trend		.022		.007		.007
LHR: all-cause mortality						
Continuous	0.32 (0.19–0.54)	<.001	0.56 (0.32–0.95)	.033	0.58 (0.37–0.91)	.018
Quartile						
Q1	Ref		Ref		Ref	
Q2	0.67 (0.51–0.89)	.006	0.72 (0.55–0.95)	.019	0.81 (0.61–1.07)	.137
Q3	0.60 (0.39–0.93)	.003	0.74 (0.58–0.96)	.022	0.81 (0.62–1.07)	.134
Q4	0.43 (0.31–0.61)	<.001	0.63 (0.46–0.87)	.005	0.67 (0.50–0.90)	.009
*P* for trend		<.001		.006		.016
LHR: cardiovascular mortality						
Continuous	0.31 (0.14–0.71)	.005	0.58 (0.24–1.38)	.217	0.6 (0.28–1.28)	.183
Quartile						
Q1	Ref		Ref		Ref	
Q2	0.68 (0.48–0.97)	.033	0.74 (0.53–1.04)	.08	0.84 (0.59–1.18)	.306
Q3	0.60 (0.39–0.93)	.023	0.71 (0.46–1.08)	.11	0.76 (0.50–1.18)	.221
Q4	0.48 (0.30–0.77)	.003	0.72 (0.44–1.17)	.187	0.76 (0.48–1.22)	.26
*P* for trend		.003		.156		.215
NHR: all-cause mortality						
Continuous	14.1 (2.60–76.6)	.002	61.1 (12.0–312)	<.001	12.7 (1.26–128)	.031
Quartile						
Q1	Ref		Ref		Ref	
Q2	1.15 (0.86–1.54)	.333	1.03 (0.77–1.37)	.86	1.03 (0.77–1.37)	.849
Q3	1.35 (0.98–1.87)	.068	1.45 (1.07–1.98)	.018	1.34 (1.00–1.79)	.047
Q4	1.51 (1.14–2.02)	.005	1.74 (1.32–2.31)	<.001	1.51 (1.12–2.04)	.007
*P* for trend		.002		<.001		.002
NHR: cardiovascular mortality						
Continuous	34.7 (2.92–413)	.005	186 (15.1–2304)	<.001	140 (6.57–2999)	.002
Quartile						
Q1	Ref		Ref		Ref	
Q2	1.18 (0.71–1.94)	.526	1.05 (0.64–1.72)	.854	1.08 (0.68–1.72)	.736
Q3	1.52 (0.97–2.40)	.069	1.66 (1.08–2.53)	.019	1.70 (1.09–2.66)	.019
Q4	1.75 (1.14–2.71)	.011	2.06 (1.35–3.15)	<.001	2.10 (1.35–3.26)	<.001
*P* for trend		.003		<.001		<.001

Model 1: no covariates were adjusted.

Model 2: adjusted for gender, age, and race.

Model 3: adjusted for gender (male or female), age (continuous), race/ethnicity (Mexican American, other Hispanic, non-Hispanic White, non-Hispanic Black, or other), education (<9th grade, 9–11th grade, high school, some college, college or above, missing data), marital status (married, widowed, divorced, separated, single, living with partner), ratio of family income to poverty (1.30, 1.31–3.50, or >3.50), BMI (continuous), SCr (continuous), WBC (continuous), ALB (continuous), TC (continuous), diabetes (yes or no), hypertension (yes or no), coronary heart disease (yes or no), smoking status (current, former, never), and drinking status (current, former, never).

CI = confidence interval, HR = hazard ratio, LHR = lymphocyte-to-high-density lipoprotein cholesterol ratio, NHR = neutrophil-to-high-density lipoprotein cholesterol ratio, UHR = uric acid-to-high-density lipoprotein cholesterol ratio.

Specifically, each 1% increase in UHR was associated with a 42% increased risk of all-cause mortality and a 2% increased risk of CVD mortality. Similarly, each 1% increase in NHR was associated with a 51% increased risk of all-cause mortality and a 110% increased risk of CVD mortality. Patients in the highest quartile (Q4) of LHR showed a significantly reduced risk of all-cause mortality (HR = 0.67, 95% CI = 0.50–0.90, *P* = .009), indicating that LHR is an independent protective factor against all-cause mortality in CHF patients.

**Figure 2.**
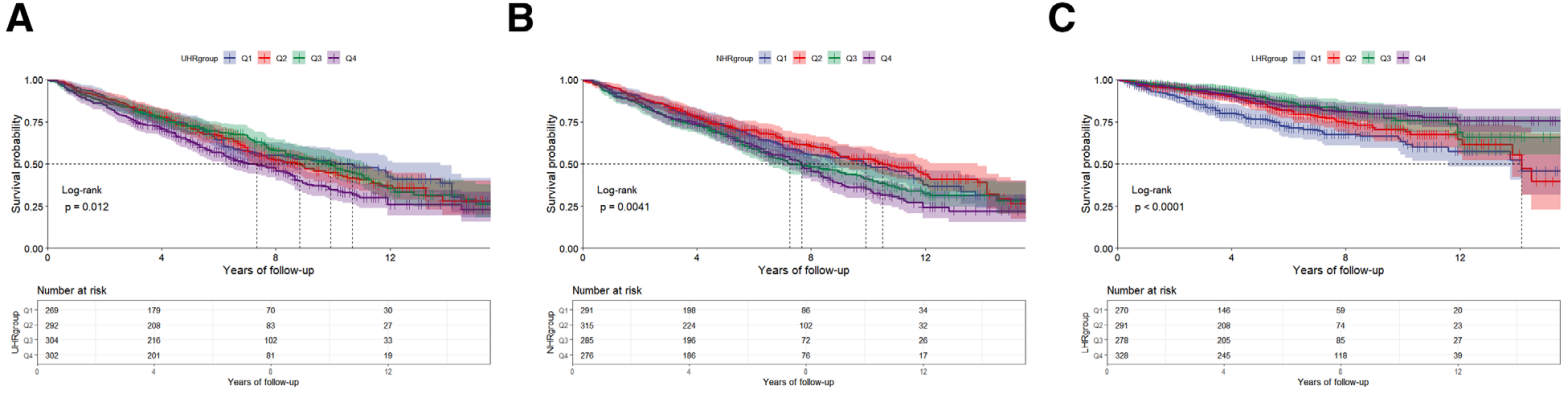
The Kaplan–Meier survival curves for the UHR associated with the risk of all-cause mortality (A). The Kaplan–Meier survival curves of the NHR with all-cause mortality (B). The Kaplan–Meier survival curve of the LHR with cardiovascular mortality (C). The y-axis shows probable survival (%). The x-axis shows the follow-up period (years). LHR = lymphocyte-to-high-density lipoprotein cholesterol ratio, NHR = neutrophil-to-high-density lipoprotein cholesterol ratio, UHR = uric acid-to-high-density lipoprotein cholesterol ratio.

Survival rates for Kaplan–Meier of all-cause mortality and CVD mortality differed among different quartiles of indices (log-rank test: *P* < .05). Patients in the higher quartiles of UHR and NHR experienced poorer survival outcomes, whereas those in the lower quartiles had better overall survival rates. While the high LHR group showed higher survival in Figures 2 and S1, Supplemental Digital Content 3.

### 3.3. Dose–response relationship among UHR, NHR, and LHR levels and mortality

Although prior multivariate Cox regression analyses identified UHR, NHR, and LHR as independent risk factors for all-cause and cardiovascular mortality in CHF patients, they did not determine specific threshold effects. To further explore potential nonlinear relationships, we applied Cox proportional hazards models with RCS and penalized smoothing splines.

**Figure 3.**
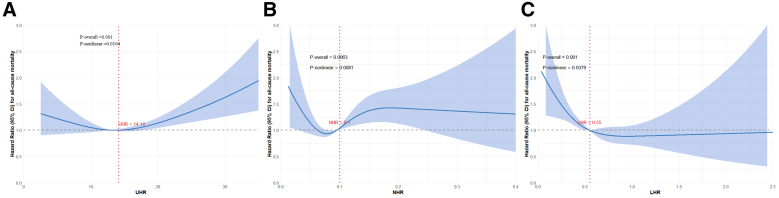
RCS curves showing the association of the UHR, NHR, and LHR with the risk of all-cause mortality (A–C) in congestive heart failure populations. LHR = lymphocyte-to-high-density lipoprotein cholesterol ratio, NHR = neutrophil-to-high-density lipoprotein cholesterol ratio, RCS = restricted cubic splines, UHR = uric acid-to-high-density lipoprotein cholesterol ratio.

Interestingly, after adjusting for age, sex, race, PIR, diabetes, angina pectoris, MI, stroke, smoking status, blood pressure, SCr, WBC, glucose, alanine aminotransferase, alkaline phosphatase, and phosphorus, we observed a nonlinear association between UHR, NHR, and LHR levels and all-cause mortality in CHF patients (*P* for nonlinearity < .05; Fig. 3). In contrast, approximately linear associations were found between UHR and LHR levels and CVD mortality (*P* for overall < .001), whereas NHR demonstrated a nonlinear relationship with CVD mortality (*P* for nonlinearity < .05; Fig. S2, Supplemental Digital Content 4).

To further explore these associations, we applied two-piecewise linear regression models to examine threshold effects of the indices on all-cause and CVD mortality (Table 3). For LHR and NHR, the inflection points for both all-cause and CVD mortality were similar, at approximately 0.1 and 0.55, respectively. However, the UHR threshold differed between the 2 outcomes: 14.14 for all-cause mortality and 13.12 for CVD mortality.

**Table 3:** Threshold effect analysis of the different indices on all-cause and cardiovascular mortality in congestive heart failure patients using the two-piecewise linear regression model.

Variables	All-cause mortality	*P* value	CVD cause mortality	*P* value
	HR* (95% CI)		HR* (95% CI)	
UHR				
Fitting model by standard linear regression	1.02 (1.00–1.03)	.013	1.03 (1.00–1.05)	.016
Fitting model by two-piecewise linear regression				
Inflection point	14.14		12.12	
UHR < 14.14	0.97 (0.94–1.01)	.13	0.99 (0.93–1.06)	.8
UHR ≥ 14.14	1.03 (1.02–1.05)	<.001	1.04 (1.01–1.06)	.008
*P* for log-likelihood ratio test	.0087		.2587	
NHR				
Fitting model by standard linear regression	5.42 (0.76–38.5)	.091	20.3 (0.77–534)	.072
Fitting model by two-piecewise linear regression				
Inflection point	0.1		0.1	
NHR < 0.1	1.14 (0.01–179)	>.9	25.8 (0.01–76,689)	.4
NHR ≥ 0.1	8.21 (0.82–82.3)	.073	19.1 (0.45–807)	.12
*P* for log-likelihood ratio test	.5133		.1509	
LHR				
Fitting model by standard linear regression	0.55 (0.39–0.77)	<.001	0.48 (0.28–0.83)	.008
Fitting model by two-piecewise linear regression				
Inflection point	0.55		0.55	
LHR < 0.55	0.22 (0.10–0.47)	<.001	0.15 (0.04–0.48)	.002
LHR ≥ 0.55	0.92 (0.56–1.50)	.7	0.97 (0.44–2.13)	>.9
*P* for log-likelihood ratio test	.0106		.0319	

Dichotomous Cox regression models adjusted for age, sex, race, PIR, diabetes, angina pectoris, myocardial infarction, stroke, smoking status, blood pressure, SCr, WBC, GLU, ALT, ALP, and phosphorus in addition to the variables themselves.

CI = confidence interval, CVD mortality = cardiovascular mortality, HR = hazard ratio, LHR = lymphocyte-to-high-density lipoprotein cholesterol ratio, NHR = neutrophil-to-high-density lipoprotein cholesterol ratio, UHR = uric acid-to-high-density lipoprotein cholesterol ratio.

* HRs are the relative risk per unit increment of UHR, NHR, and LHR.

When UHR exceeded 14.14, each unit increase was associated with a 3% increase in the adjusted HR for all-cause mortality (HR = 1.03, 95% CI = 1.02–1.05, log-likelihood ratio test *P* = .0087), and a 4% increase in adjusted HR for CVD mortality (HR = 1.04, 95% CI = 1.01–1.06, *P* < .05). Conversely, for LHR, when values were >0.55, each unit decrease was associated with an 88% reduction in adjusted HR for all-cause mortality (HR = 0.22, 95% CI = 0.10–0.47, *P* < .05) and an 85% reduction in adjusted HR for CVD mortality (HR = 0.15, 95% CI = 0.04–0.48, *P* < .05). Notably, no significant threshold association was observed between NHR and either all-cause or cardiovascular mortality.

### 3.4. Subgroup analysis

**Figure 4.**
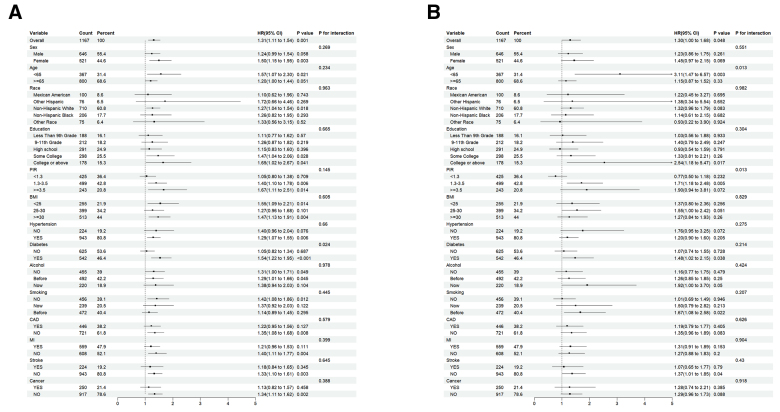
Subgroup analyses of the association between the UHR index and mortality in congestive heart failure patients. (A) All-cause mortality. (B) Cardiovascular mortality. When analyzing subgroup variables, age, sex, race, education, PIR, smoking status, alcohol, BMI, CAD, MI, stroke, cancer, diabetes, and hypertension were adjusted in addition to the variables themselves. BMI = body mass index, CAD = coronary artery disease, CI = confidence interval, CVD = cardiovascular disease, HR = hazard ratio, MI = myocardial infarction, PIR = poverty-to-income ratio, UHR = uric acid-to-high-density lipoprotein cholesterol ratio.

Subgroup analyses were conducted to assess whether the association between UHR and all-cause mortality was consistent across different demographic and clinical subgroups in patients with CHF. Based on the threshold identified by RCS analysis, participants were classified into high-UHR (≥14.14) and low-UHR (<14.14) groups. The results of interaction tests and subgroup analyses, stratified by age, gender, education level, race, PIR, BMI, diabetes, hypertension, coronary artery disease (CAD), MI, stroke, cancer, smoking status, and alcohol consumption, are presented in Figures 4 and S3 and S4, Supplemental Digital Content 5.

In the overall population, the high-UHR group showed a 31% increased risk of all-cause mortality compared with the low-UHR group. The CI did not cross the null line, indicating statistical significance. The association between high UHR and all-cause mortality was more prominent in patients with diabetes. For cardiovascular mortality, stronger associations were observed in individuals younger than 65 years and in those with PIR values between 1.3 and 3.5. Specifically, among CHF patients with diabetes, each 1-unit increase in UHR (when UHR ≥ 14.14) was associated with a 54% increase in all-cause mortality and a 71% increase in cardiovascular mortality. In individuals younger than 65 years and those with PIR between 1.3 and 3.5, the risk of cardiovascular mortality increased by 211% per unit increase in UHR (*P* for interaction = .013).

Similarly, when NHR was 0.1 or higher, the risk of all-cause mortality increased with higher PIR levels (*P* for interaction = .043). The high NHR group consistently showed a survival disadvantage across all subgroups, as shown in Figure S3, Supplemental Digital Content 5. In contrast, the high LHR group demonstrated a consistent survival advantage in each subgroup, as shown in Figure S4, Supplemental Digital Content 6.

These findings indicate that the associations of UHR, NHR, and LHR with mortality in CHF patients are generally stable and reliable across diverse subgroups.

### 3.5. The predictive ability of UHR, NHR, and LHR level for mortality in HF

ROC curve analysis was conducted to evaluate the predictive value of the composite indices UHR, NHR, and LHR for all-cause and cardiovascular mortality in patients with CHF.

**Figure 5.**
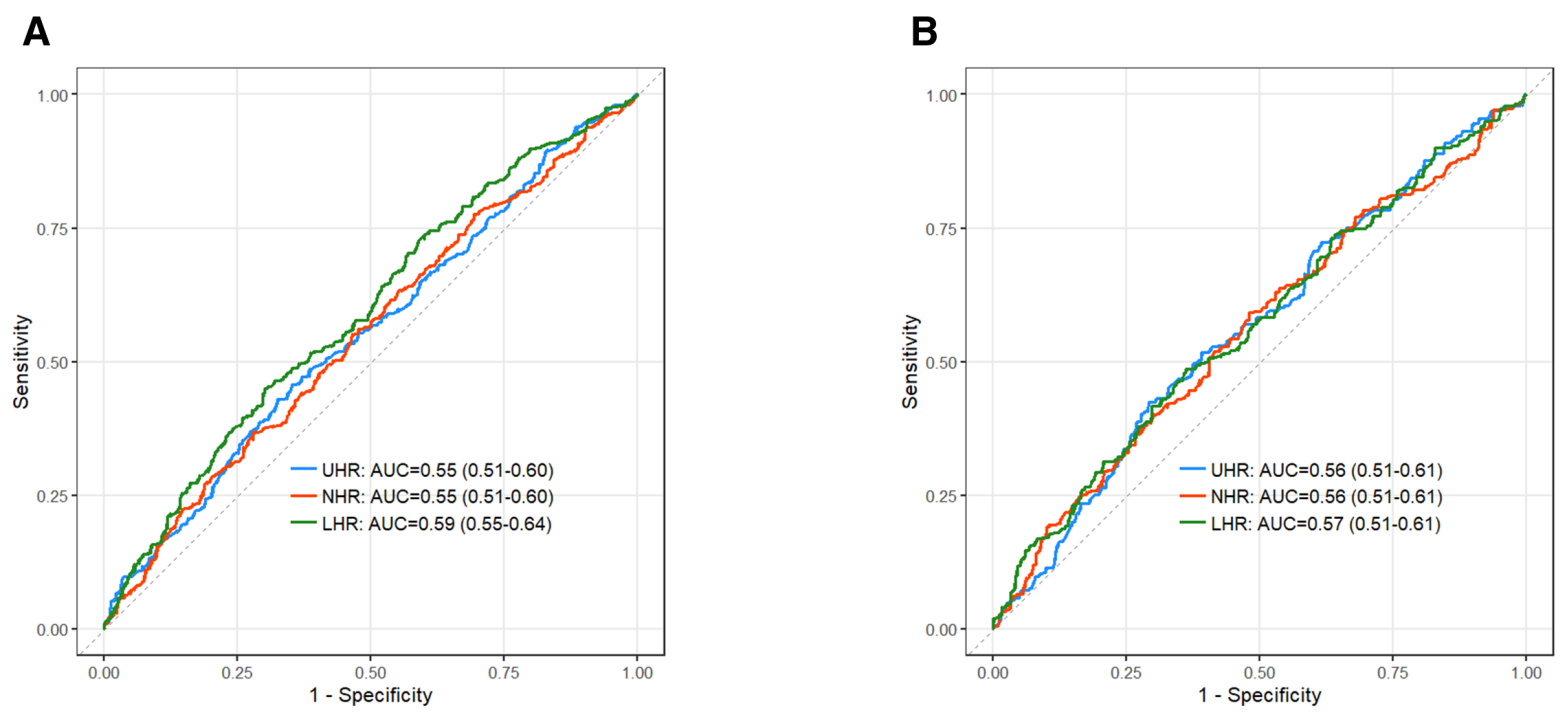
ROC curves and AUCs values (with a 95% confidence band) of the UHR, NHR, and LHR levels for predicting all-cause mortality (A) and cardiovascular mortality (B). Adjusting for gender, age, race/ethnicity, education, marital status, PIR, BMI, SCr, WBC, ALB, TC, diabetes, hypertension, CAD, smoking status, and drinking status. ALB = albumin, AUC = areas under the curve, BMI = body mass index, CAD = coronary artery disease, LHR = lymphocyte-to-high-density lipoprotein cholesterol ratio, NHR = neutrophil-to-high-density lipoprotein cholesterol ratio, PIR = poverty-to-income ratio, ROC = receiver operating characteristic, SCr = serum creatinine, TC = total cholesterol, UHR = uric acid-to-high-density lipoprotein cholesterol ratio, WBC = white blood cell.

**Figure 6.**
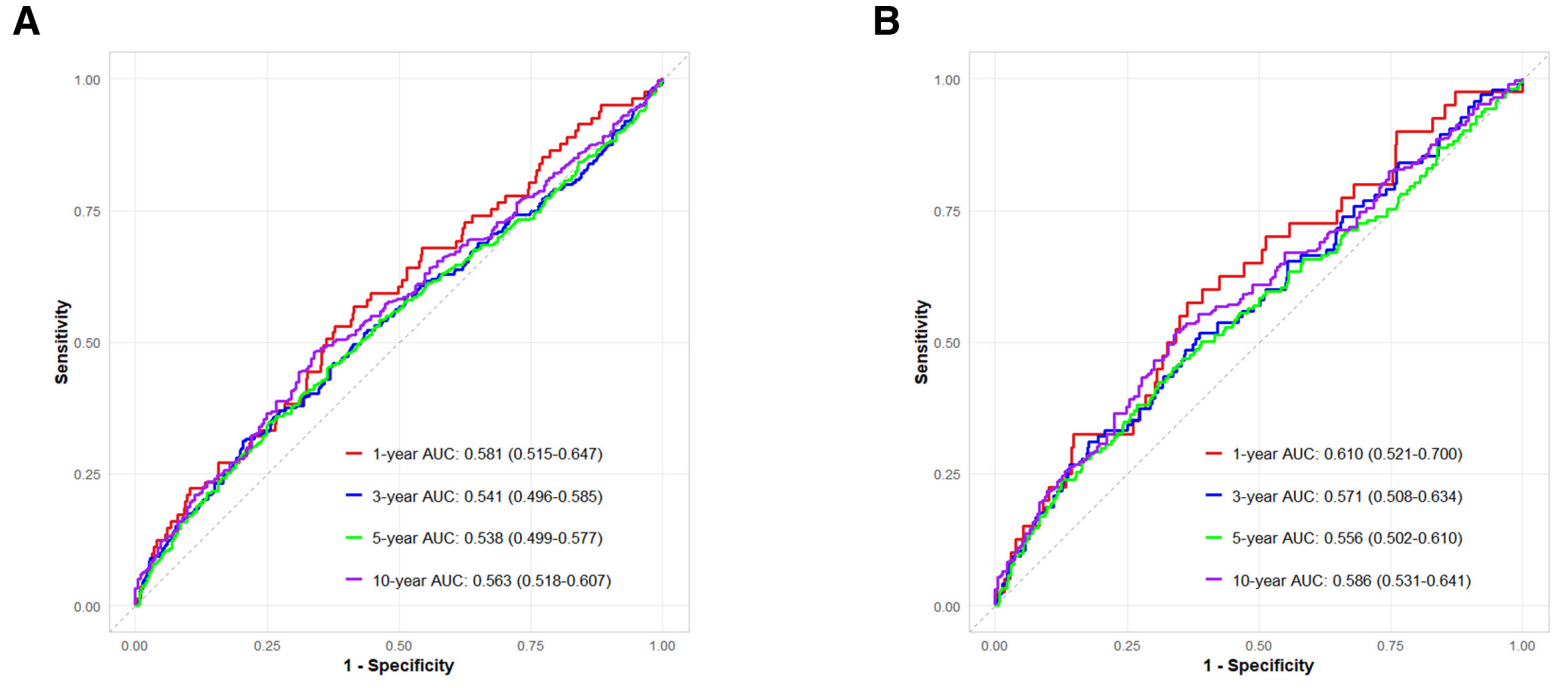
Time-dependent ROC curves and time-dependent AUCs values of the UHR level for predicting all-cause mortality (A) and cardiovascular mortality (B). Adjusting for gender, age, race/ethnicity, education, marital status, PIR, BMI, SCr, WBC, ALB, TC, diabetes, hypertension, CAD, smoking status, and drinking status. ALB = albumin, AUC = areas under the curve, BMI = body mass index, CAD = coronary artery disease, PIR = poverty-to-income ratio, ROC = receiver operating characteristic, SCr = serum creatinine, TC = total cholesterol, UHR = uric acid-to-high-density lipoprotein cholesterol ratio, WBC = white blood cell.

As shown in Figure 5A, the area under the curve (AUC) values for UHR, NHR, and LHR in predicting all-cause mortality were 0.55, 0.55, and 0.59, respectively. For cardiovascular mortality (Fig. 5B), the corresponding AUC values were 0.56, 0.56, and 0.57. Time-dependent ROC curves were used to evaluate the predictive performance of UHR levels for mortality. After adjusting for gender, age, race/ethnicity, education, marital status, PIR, BMI, SCr, WBC, ALB, TC, diabetes, hypertension, CAD, smoking status, and drinking status, the AUC values for predicting all-cause mortality at 1, 3, 5, and 10 years were 0.581, 0.541, 0.538, and 0.563, respectively. For CVD mortality, the AUC values were 0.610, 0.571, 0.556, and 0.586 (Fig. 6). These findings suggest that the predictive value of UHR for mortality gradually declined over time. Time-dependent ROC analyses for NHR and LHR levels showed similar patterns and are presented in Figure S5, Supplemental Digital Content 7. A summary of the AUC values with 95% CIs for the ROC and time-dependent ROC curves is provided in Table S4, Supplemental Digital Content 8.

### 3.6. Assessment of clinical utility and predictive accuracy of multivariate Cox regression models using DCA and calibration curves

**Figure 7.**
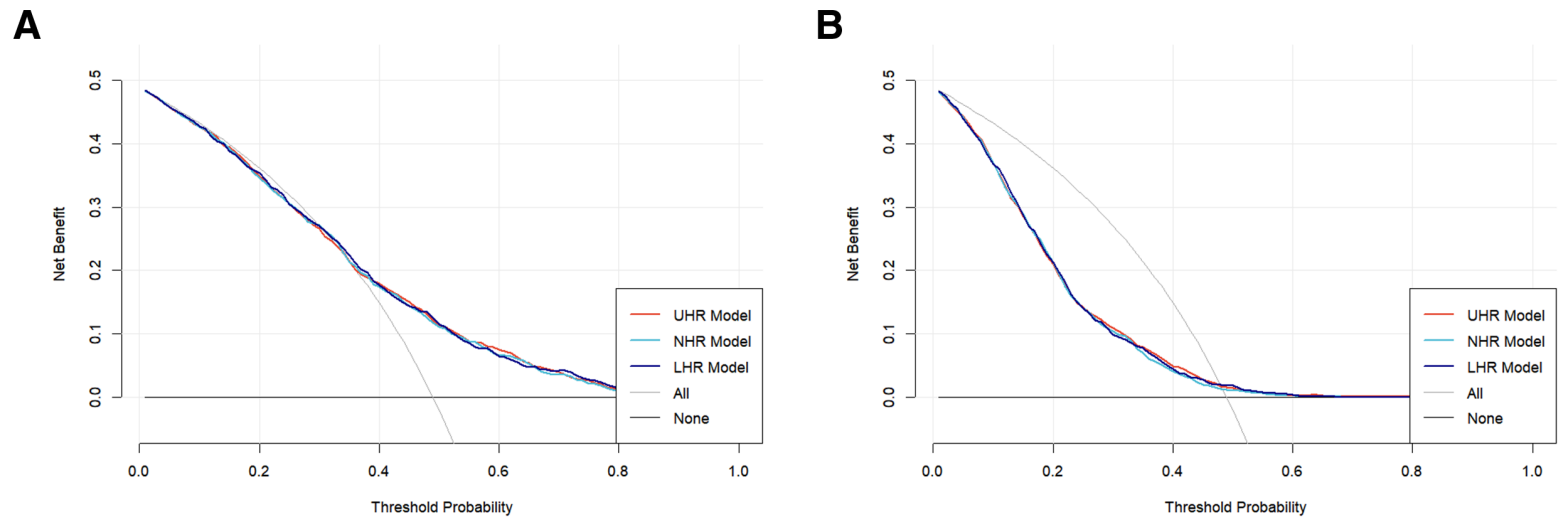
Assessing the clinical utility of the UHR, NHR, and LHR model through decision curve analysis in all-cause mortality (A) and cardiovascular mortality (B) among congestive heart failure patients. LHR = lymphocyte-to-high-density lipoprotein cholesterol ratio, NHR = neutrophil-to-high-density lipoprotein cholesterol ratio, UHR = uric acid-to-high-density lipoprotein cholesterol ratio.

Multivariate Cox Model 3, constructed to evaluate the associations of UHR, NHR, and LHR with all-cause and cardiovascular mortality in CHF patients, demonstrated similar areas under the DCA curves for predicting all-cause mortality. These areas were notably higher than those of both the unadjusted and fully adjusted models, indicating greater net benefit across a wide range of threshold probabilities. For cardiovascular mortality prediction, the DCA areas of the 3 models exceeded those of the unadjusted model but were lower than those of the fully adjusted model, suggesting limited net benefit in this context. These results support the clinical utility of Cox Model 3 in predicting all-cause and CVD mortality based on UHR, NHR, and LHR in patients with CHF (Fig. 7).

**Figure 8.**
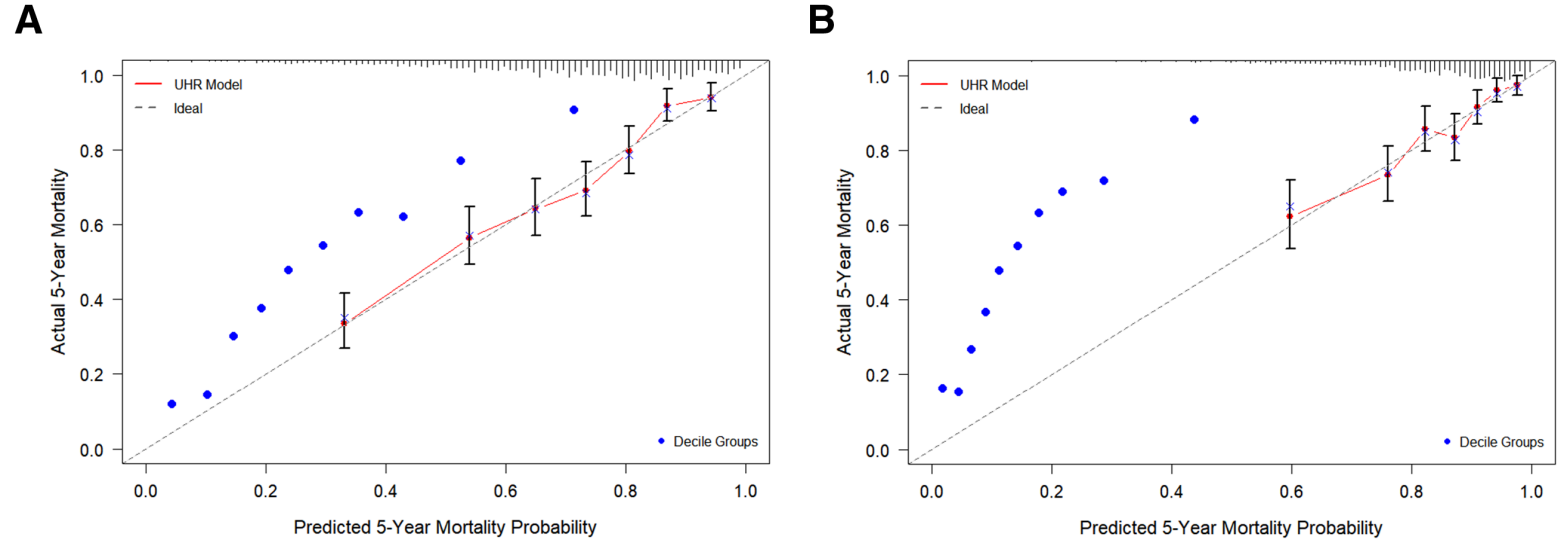
Calibration curves of the Cox model predictions in congestive heart failure patients. (A) Calibration curve of the Cox regression model for predicting all-cause mortality with UHR. (B) Calibration curve of the Cox regression model for predicting cardiovascular mortality with UHR. UHR = uric acid-to-high-density lipoprotein cholesterol ratio.

The calibration curves further indicate that when the predicted probability exceeds 0.36, the overall multivariate Cox model provides reliable estimates of all-cause mortality, with the prediction curve closely aligning with the 45° reference line. However, when patients were divided into 10 equal groups based on ascending predicted risk, the corresponding calibration points consistently lay above the reference line, suggesting a tendency of the model to underestimate risk (Figs. 8 and S6, Supplemental Digital Content 9).

### 3.7. Interpretability and applications of models via SHAP

**Figure 9.**
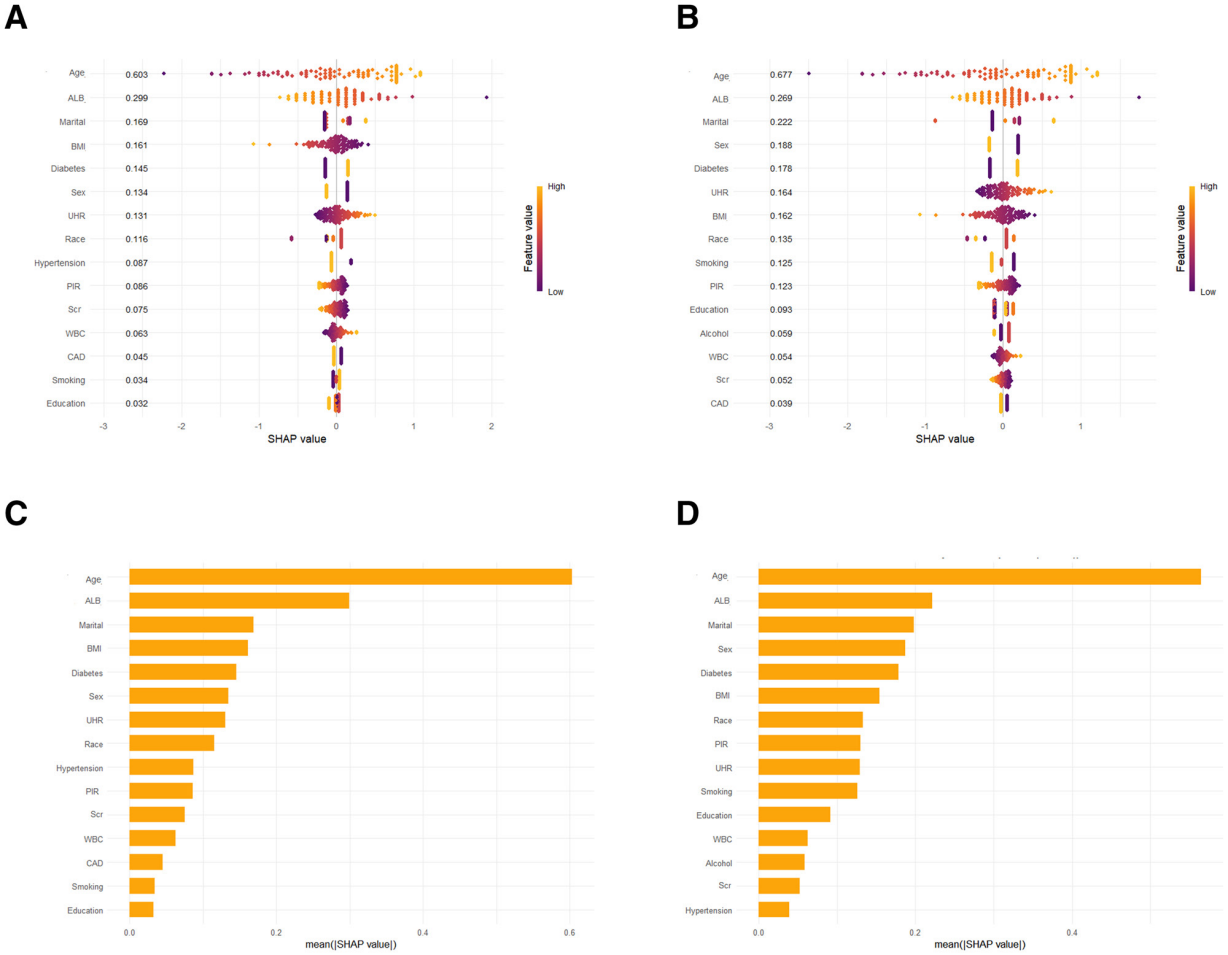
SHAP analysis of the UHR for predicting all-cause and cardiovascular mortality in patients with CHF. (A) Beeswarm plot of SHAP values for the UHR and other clinical features in predicting all-cause mortality. Yellow and purple dots indicate high and low feature values, respectively (yellow: high values; purple: low values), while the horizontal position reflects directional impact on prediction outcomes (rightward: increased risk; leftward: decreased risk). (B) SHAP beeswarm plot for cardiovascular mortality prediction with consistent color coding. (C) Feature importance ranking based on mean absolute SHAP values for all-cause mortality prediction. (D) Feature importance ranking based on mean absolute SHAP values for cardiovascular mortality prediction. CHF = congestive heart failure, SHAP = SHapley Additive exPlanations, UHR = uric acid-to-high-density lipoprotein cholesterol ratio.

SHAP analysis revealed the contributions of individual features in the multivariate Cox regression model predicting all-cause and cardiovascular mortality among CHF patients. Age, albumin level, BMI, diabetes status, sex, and UHR emerged as the most influential predictors for all-cause mortality. Specifically, increased age, lower albumin levels, higher BMI, presence of diabetes, male sex, and elevated UHR were all significantly associated with a higher risk of all-cause mortality. The variable importance ranking further identified age, albumin, marital status, BMI, diabetes, sex, race, and UHR as the top 8 contributors to the model’s predictive performance (Fig. 9). Similar SHAP analyses for models based on NHR and LHR demonstrated consistent patterns, underscoring the robust predictive value of these combined indices for both all-cause and cardiovascular mortality in CHF patients (Figs. S7 and S8, Supplemental Digital Content 10). These findings highlight the potential of UHR, NHR, and LHR as important prognostic biomarkers in HF risk stratification.

## 4. Discussion

In this study, we analyzed data from the NHANES database, including 1167 participants with HF, to investigate associations between composite indices, specifically UHR, NHR, and LHR, and patient prognosis. Significant correlations were observed between these indices and both all-cause and cardiovascular mortality. Subgroup analyses and interaction tests confirmed consistent patterns across various populations. Furthermore, smoothing curve fitting and threshold effect analyses suggested potential nonlinear associations between UHR, NHR, LHR, and HF outcomes.

Recent NHANES-based studies on HF have primarily focused on the relationships among physical activity, inflammatory markers, and insulin levels.^[33,34]^ Prior research has also examined the associations between HDL-C and SUA with cardiovascular disease and HF, respectively. Early investigations demonstrated that higher HDL-C levels are associated with reduced cardiovascular disease incidence.^[35]^ Consistent with these findings, several studies reported an inverse relationship between HDL-C levels and cardiovascular event risk.^[36–38]^ These observations align with our finding that elevated UHR, reflecting low HDL-C levels, correlates positively with cardiovascular and all-cause mortality in CHF patients. HDL-C exerts anti-inflammatory effects by removing cholesterol from macrophages, thus reducing inflammation.^[36]^ However, the protective role of HDL-C, particularly in HF prognosis, has been challenged by recent large-scale studies, indicating that the association may be neutral or more complex.^[39,40]^ Consistent with these findings, our data show that HDL-C level alone was not significantly associated with mortality in patients with CHF, irrespective of covariate adjustment.

Elevated SUA has been linked to increased cardiovascular and all-cause mortality in patients with cardiovascular diseases. An NHANES study highlighted that this association is more pronounced in male HF patients, with each 1 mg/dL increase in uric acid (UA) corresponding to an 11% increase in all-cause mortality risk and an 18% increase in cardiovascular mortality risk.^[41]^ Although the mechanisms connecting hyperuricemia and HF mortality are not fully understood, several pathways have been proposed. UA deposits on vascular walls may impair nitric oxide production and vascular dilation, increase oxidative stress, and promote pro-inflammatory factors, leading to endothelial dysfunction. In addition, hyperuricemia can enhance low-density lipoprotein oxidation and the generation of reactive oxygen species, contributing to atherosclerosis.^[42–44]^ UA also activates the NLRP3 inflammasome in macrophages, leading to interleukin-1 beta production. This cytokine subsequently acts on tissue-resident cells to activate nuclear factor-kappa B signaling, thereby amplifying the inflammatory cascade.^[13,45]^ In summary, elevated UA levels promote inflammation, leading to vascular endothelial dysfunction, oxidative stress, and various inflammatory responses.^[46,47]^ Moreover, SUA levels have been associated with left ventricular remodeling in chronic ischemic HF.^[48]^

The combination of inflammatory and anti-inflammatory markers has been demonstrated as a robust predictor of mortality in patients with HFpEF.^[49]^ Compared to HFrEF, inflammatory markers and related comorbidities exhibit stronger associations with HF risk in HFpEF. This study applied indices related to HDL-C, including UHR, NHR, and LHR, offering a more comprehensive and direct assessment of cardiovascular and all-cause mortality risk in CHF. While the precise biological mechanisms underlying the associations between these indices and mortality remain unclear, changes in UHR, NHR, and LHR may reflect metabolic imbalances that exacerbate HF progression. Elevated UA, neutrophil counts, reduced lymphocytes, and low HDL-C may synergistically promote disease advancement. Prior research suggests that combining HDL-C and SUA measurements improves cardiovascular risk prediction compared to either marker alone.^[20]^ Notably, some studies have reported that UHR outperforms UA or HDL-C alone in predicting CAD in certain populations.^[50]^ However, ROC analyses revealed that UHR, NHR, and LHR individually exhibited modest predictive ability for mortality, with AUC values ranging from 0.55 to 0.59. While these associations reached statistical significance, their modest discriminatory capacity warrants consideration. Several factors may account for this finding. First, the opposing physiological effects of UA and neutrophils versus HDL-C on mortality risk may partially offset each other within these composite indices, thereby attenuating their individual predictive performance.^[16–18]^ Second, each ratio captures only a single dimension of the complex pathophysiological landscape in HF, which inherently limits its discriminatory power when used in isolation. Third, the heterogeneity of the CHF population, including unmeasured factors such as HF subtype, severity, and medication regimens, may have diluted the predictive signal. Despite these limitations, the combined indices provided more balanced and robust risk assessment after multivariate adjustment, suggesting their potential utility as complementary tools in clinical risk stratification. The observed AUC values are consistent with those reported for other novel biomarkers in population-based studies, reflecting the multifactorial nature of mortality risk in chronic disease populations.^[51]^ Nevertheless, given the modest predictive performance, these ratios should be interpreted cautiously and are best used in conjunction with established clinical risk factors rather than as standalone predictors.

Our study yielded 7 key findings. First, after adjusting for confounders, elevated UHR and NHR were independent risk factors for cardiovascular and all-cause mortality, whereas LHR was protective in CHF patients. Second, Kaplan–Meier analyses showed that higher UHR and NHR corresponded to increased cumulative mortality, while higher LHR was associated with reduced mortality over time. Third, restrictive cubic spline analyses revealed a J-shaped association between UHR and all-cause mortality, with a threshold at 14.14; above this, UHR was an independent mortality risk factor. LHR exhibited an L-shaped association, and NHR showed nonlinear correlations with mortality outcomes. Fourth, UHR’s prognostic value for all-cause mortality was particularly pronounced in diabetic patients, whereas its association with cardiovascular mortality was stronger among individuals younger than 65 years and those with moderate poverty-to-income ratios. These subgroup patterns may relate to metabolic disturbances and inflammatory status in diabetes and socioeconomic factors influencing cardiovascular risk. In diabetic patients, UA production is increased while excretion is decreased. In contrast, the levels of HDL-C tend to be reduced, thus showing a significant correlation with all-cause mortality.^[28]^ More notably, patients aged <65 years who have better physical functions and fewer comorbidities are more sensitive to metabolic and inflammatory damage, making them more prone to cardiovascular events. Fifth, time-dependent ROC analyses indicated that UHR predicted early mortality, while NHR’s predictive value increased over time, and LHR’s predictive capacity fluctuated, suggesting stage-specific pathophysiological roles. Sixth, DCA suggested that prediction models based on these indices provided clinical benefit within moderate risk thresholds for all-cause mortality but showed limited benefit for cardiovascular mortality, possibly due to confounding factors and the complexity of cardiovascular-specific risks. Finally, SHAP analysis highlighted age, sex, albumin level, and marital status as dominant predictors of mortality, with UHR, NHR, and LHR contributing but ranked lower, likely reflecting their biomarker-specific roles limited by disease stage and sample size.

These findings reinforce the concept that UHR, NHR, and LHR collectively capture interactions among metabolism, inflammation, immunity, and HF, providing valuable tools for cardiovascular risk assessment. Previous studies have demonstrated UHR as a reliable marker of cardiovascular risk across various conditions including acute MI and ischemic heart disease.^[52–54]^ Similarly, NHR has been linked to coronary artery lesion severity in acute coronary syndrome,^[55]^ while research on LHR remains limited. However, this study utilized cross-sectional NHANES data to identify UHR, NHR, and LHR as novel predictors of all-cause and cardiovascular mortality in CHF. Multivariate Cox regression and subgroup analyses enhanced the robustness of the findings. Our results extend these observations to CHF, suggesting that these indices may reflect underlying chronic low-grade inflammation, oxidative stress, and comorbid conditions influencing mortality risk.

The UHR, NHR, and LHR are affordable and readily accessible biomarkers with substantial clinical value for risk stratification and community-level screening in CHF. Derived from routine laboratory parameters, these composite markers reflect interconnected pathophysiological processes, including purine metabolism, inflammatory activation, and immune response, providing an integrated assessment of mortality risk. In our analysis of NHANES 2003 to 2016 data, UHR, NHR, and LHR showed significant associations with mortality in CHF, supporting their utility in identifying high-risk patients who may benefit from intensified treatment strategies. By transforming commonly available laboratory results into meaningful prognostic information, these ratios can be easily incorporated into standard clinical evaluation workflows.

Despite advances in treatment, the prognosis of CHF remains poor. Chronic inflammation and metabolic dysfunction play central roles in disease progression, yet anti-inflammatory therapies have shown limited success to date.^[56]^ Our findings suggest that targeting metabolic-inflammatory pathways, reflected by UHR, NHR, and LHR, may aid early risk stratification and intervention. Several limitations should be considered. First, the cross-sectional design precludes causal inference and cannot exclude residual confounding. Second, only baseline biomarker levels were assessed; thus, the prognostic value of their longitudinal changes remains unknown. Third, CHF diagnosis relied on self-report. While validated and widely used in NHANES studies, this approach lacks objective evidence such as natriuretic peptide levels or echocardiographic findings, and may therefore introduce misclassification bias. Fourth, critical clinical data for HF phenotyping are unavailable in NHANES. This includes left ventricular ejection fraction, which prevents differentiation between HFrEF and HFpEF, a distinction with important prognostic and therapeutic implications. In addition, New York Heart Association functional class and specific indications for prescribed medications are not collected, limiting adjustment for these factors. Fifth, the 2003 to 2016 NHANES cycles were selected as they provide the most complete harmonized dataset for all variables of interest. Despite these limitations, key strengths include the large, nationally representative sample, standardized data collection, and comprehensive evaluation of novel biomarkers.

In summary, this study demonstrates that UHR, NHR, and LHR are significant predictors of all-cause and cardiovascular mortality in patients with CHF. These indices reflect important interactions between metabolic, inflammatory, and immune factors relevant to HF prognosis. Incorporating UHR, NHR, and LHR into risk assessment may enhance the evaluation of patient outcomes in this population. However, the inherent limitations of the NHANES database and the modest predictive performance observed must be acknowledged. Future large-scale prospective cohort studies incorporating dynamic assessments of UHR, NHR, and LHR are warranted to validate our findings and elucidate the underlying biological mechanisms, thereby improving their clinical utility and ultimately patient outcomes.

## Acknowledgments

All authors thank the participants and investigators in the NHANES study.

## Author contributions

**Conceptualization:** Xinxin Wang.

**Data curation:** Xinxin Wang.

**Validation:** Xinxin Wang.

**Visualization:** Xinxin Wang.

**Writing – original draft:** Xinxin Wang.

**Writing – review & editing:** Yongming Liu.
























