# Supplementary material for: The uric acid-to-high-density lipoprotein cholesterol ratio, neutrophil-to-high-density lipoprotein cholesterol ratio, and lymphocyte-to-high-density lipoprotein cholesterol ratio as risk indicators for mortality in congestive heart failure: A cross-sectional analysis of NHANES 2003 to 2016
Source: Medicine (Baltimore). 2026 Jun 26;105(26):e49313. doi: 10.1097/MD.0000000000049313 (PMC13313641; doi:10.1097/MD.0000000000049313)
Supplement: Supplementary file 1 [file medi-105-e49313-s001.docx]

**Supplementary Tables**

**Table S1 Clinical characteristics of the study participants based on** neutrophil to high-density lipoprotein cholesterol ratio **index quartiles**

| **Characteristic** | **N** | | **Overall** | **Q1(<0.0681)** | **Q2(0.0681-0.0979)** | | **Q3(0.0979-0.1342)** | **Q4(>0.1342)** | | **p-value** |
| --- | --- | --- | --- | --- | --- | --- | --- | --- | --- | --- |
|  | |  | N = 1167 | N = 291 | N =315 | N = 285 | | N =276 |  | |
| Sex | | 1,167 |  |  |  |  | |  | 0.045 | |
| Male | |  | 646(54%) | 143(50%) | 156(47%) | 172(60%) | | 175(59%) |  | |
| Female | |  | 521(46%) | 148(50%) | 159(53%) | 113(40%) | | 101(41%) |  | |
| Age, years | | 1,167 | 67±(12) | 67±(13) | 69±(11) | 67±(12) | | 66±(12) | 0.01 | |
| Race | | 1,167 |  |  |  |  | |  | 0.001 | |
| Mexican | |  | 100(3.0%) | 14(1.4%) | 29(3.8%) | 29(3.3%) | | 28(3.7%) |  | |
| American | |  | 76(2.8%) | 15(2.1%) | 34(5.0%) | 14(2.5%) | | 13(1.7%) |  | |
| Other Hispanic | |  | 710(78%) | 151(73%) | 179(75%) | 184(83%) | | 196(84%) |  | |
| Non-Hispanic | |  | 206(9.1%) | 90(15%) | 52(9.8%) | 42(7.4%) | | 22(4.1%) |  | |
| White | |  | 75(6.6%) | 21(8.6%) | 21(6.7%) | 16(4.2%) | | 17(7.1%) |  | |
| Education | | 1,167 |  |  |  |  | |  | 0.014 | |
| Less Than 9th Grade | |  | 188(10%) | 39(7.6%) | 56(11%) | 45(9.7%) | | 48(13%) |  | |
| 9-11th Grade | |  | 212(16%) | 52(15%) | 50(15%) | 54(18%) | | 56(19%) |  | |
| High School Grad/GED | |  | 291(26%) | 58(20%) | 73(24%) | 85(31%) | | 75(28%) |  | |
| Some College | |  | 298(29%) | 87(35%) | 72(24%) | 69(30%) | | 70(28%) |  | |
| College Graduate or above | |  | 178(19%) | 55(23%) | 64(27%) | 32(12%) | | 27(13%) |  | |
| Marital | | 1,167 |  |  |  |  | |  | 0.6 | |
| Married | |  | 605(55%) | 139(55%) | 173(57%) | 148(54%) | | 145(57%) |  | |
| Widowed | |  | 276(20%) | 78(22%) | 64(17%) | 76(23%) | | 58(18%) |  | |
| Divorced | |  | 141(11%) | 29(9.5%) | 37(11%) | 30(9.5%) | | 45(15%) |  | |
| Separated | |  | 34(2.6%) | 9(1.7%) | 9(2.5%) | 10(3.3%) | | 6(2.8%) |  | |
| Single | |  | 74(6.8%) | 26(6.4%) | 22(9.7%) | 13(7.4%) | | 13(3.6%) |  | |
| Living with partner | |  | 37(3.9%) | 10(5.7%) | 10(3.2%) | 8(3.3%) | | 9(3.6%) |  | |
| PIR | | 1,167 |  |  |  |  | |  | 0.5 | |
| <1.30 | |  | 425(27%) | 94(20%) | 119(28%) | 106(28%) | | 106(30%) |  | |
| 1.31-3.50 | |  | 499(46%) | 130(50%) | 125(43%) | 123(44%) | | 121(46%) |  | |
| ≥3.50 | |  | 243(28%) | 67(30%) | 71(29%) | 56(29%) | | 49(24%) |  | |
| SBP, mmHg | | 1,167 | 129±(20) | 128±(20) | 130±(19) | 129±(20) | | 127±(21) | 0.2 | |
| DBP, mmHg | | 1,167 | 70±(16) | 71±(16) | 70±(16) | 69±(18) | | 69±(14) | 0.2 | |
| BMI, kg/m2 | | 1,167 | 30±(7) | 28±(6) | 30±(7) | 31±(7) | | 33±(7) | <0.001 | |
| WAIST, cm | | 1,167 | 107±(17) | 98±(14) | 107±(16) | 109±(17) | | 114±(16) | <0.001 | |
| HbA1c, % | | 1,167 | 6.24±(1.27) | 5.73±(0.70) | 6.23±(1.27) | 6.24±(1.23) | | 6.77±(1.54) | <0.001 | |
| FBG, mmol/L | | 1,167 | 6.81±(2.44) | 5.87±(1.29) | 6.62±(2.45) | 7.02±(2.44) | | 7.76±(2.92) | <0.001 | |
| TC, mg/dL | | 1,167 | 177±(45) | 184±(43) | 179±(47) | 172±(44) | | 173±(44) | 0.029 | |
| LDL, mg/dL | | 1,167 | 193±(256) | 167±(170) | 200±(255) | 199±(259) | | 206±(318) | 0.3 | |
| HDL, mg/dL | | 1,167 | 50±(16) | 65±(18) | 51±(12) | 45±(11) | | 37±(9) | <0.001 | |
| TG, mg/dL | | 1,167 | 153±(128) | 101±(59) | 141±(92) | 161±(96) | | 212±(196) | <0.001 | |
| SUA, mg/dL | | 1,167 | 6.15±(1.71) | 5.68±(1.52) | 6.03±(1.56) | 6.28±(1.76) | | 6.60±(1.86) | <0.001 | |
| ALP, IU/L | | 1,167 | 74±(28) | 76±(33) | 72±(30) | 72±(25) | | 76±(24) | 0.13 | |
| ALT, IU/L | | 1,167 | 25±(42) | 24±(13) | 24±(14) | 24±(15) | | 27±(81) | >0.9 | |
| AST, IU/L | | 1,167 | 26±(20) | 29±(15) | 26±(12) | 24±(7) | | 26±(35) | 0.002 | |
| ALB, g/L | | 1,167 | 4.13±(0.34) | 4.15±(0.36) | 4.19±(0.33) | 4.11±(0.35) | | 4.05±(0.32) | <0.001 | |
| TBil, mmol/L | | 1,167 | 12.2±(5.4) | 12.5±(5.6) | 12.7±(5.9) | 12.2±(5.3) | | 11.5±(4.6) | 0.3 | |
| LDH, IU/L | | 1,167 | 140±(33) | 144±(29) | 138±(32) | 140±(34) | | 139±(38) | 0.027 | |
| P, mmol/L | | 1,167 | 1.22±(0.19) | 1.22±(0.19) | 1.22±(0.21) | 1.22±(0.18) | | 1.21±(0.20) | 0.7 | |
| SCr, umol/L | | 1,167 | 9,864±(6,124) | 9,578±(5,868) | 9,333±(5,708) | 9,833±(6,670) | | 10,730±(6,137) | 0.14 | |
| BUN, mg/dL | | 1,167 | 19±(10) | 18±(8) | 18±(8) | 20±(11) | | 21±(12) | 0.007 | |
| WBC,1000cells/uL | | 1,167 | 7.68±(2.53) | 5.81±(1.29) | 6.87±(1.47) | 8.23±(2.90) | | 9.82±(2.10) | <0.001 | |
| Lymphocyte,1000cells/uL | | 1,167 | 26±(9) | 31±(9) | 27±(8) | 24±(9) | | 21±(7) | <0.001 | |
| Mono,1000cells/uL | | 1,167 | 8.53±(2.45) | 9.65±(2.79) | 8.82±(2.27) | 8.10±(1.96) | | 7.52±(2.20) | <0.001 | |
| Neutrophil,1000cells/uL | | 1,167 | 4.77±(1.80) | 3.20±(0.85) | 4.12±(0.98) | 5.18±(1.29) | | 6.63±(1.80) | <0.001 | |
| PLT,1000cells/uL | | 1,167 | 222±(73) | 211±(76) | 211±(65) | 224±(70) | | 242±(78) | <0.001 | |
| Alcohol | | 1,167 |  |  |  |  | |  | 0.12 | |
| Never | |  | 455(33%) | 117(33%) | 130(36%) | 114(33%) | | 94(29%) |  | |
| Former | |  | 492(47%) | 114(43%) | 121(42%) | 127(53%) | | 130(50%) |  | |
| Current | |  | 220(20%) | 60(24%) | 64(22%) | 44(14%) | | 52(21%) |  | |
| Smoking | | 1,167 |  |  |  |  | |  | <0.001 | |
| Never | |  | 456(39%) | 142(48%) | 145(45%) | 90(30%) | | 79(32%) |  | |
| Former | |  | 239(22%) | 49(21%) | 34(11%) | 74(27%) | | 82(31%) |  | |
| Current | |  | 472(39%) | 100(31%) | 136(45%) | 121(44%) | | 115(37%) |  | |
| Hypertension | | 1,167 | 943(76%) | 235(76%) | 259(79%) | 221(75%) | | 228(75%) | 0.8 | |
| Diabetes | | 1,167 | 542(44%) | 90(23%) | 141(44%) | 140(47%) | | 171(61%) | <0.001 | |
| CAD | | 1,167 |  |  |  |  | |  | 0.006 | |
| yes | |  | 446(34%) | 89(26%) | 112(31%) | 121(40%) | | 124(41%) |  | |
| no | |  | 721(66%) | 202(74%) | 203(69%) | 164(60%) | | 152(59%) |  | |
| AP | | 1,167 |  |  |  |  | |  | 0.027 | |
| yes | |  | 333(30%) | 64(26%) | 84(26%) | 103(40%) | | 82(29%) |  | |
| no | |  | 834(70%) | 227(74%) | 231(74%) | 182(60%) | | 194(71%) |  | |
| MI | | 1,167 |  |  |  |  | |  | 0.2 | |
| yes | |  | 559(47%) | 120(44%) | 144(43%) | 142(49%) | | 153(54%) |  | |
| no | |  | 608(53%) | 171(56%) | 171(57%) | 143(51%) | | 123(46%) |  | |
| Stroke | | 1,167 |  |  |  |  | |  | 0.7 | |
| yes | |  | 224(17%) | 55(16%) | 60(16%) | 57(20%) | | 52(17%) |  | |
| no | |  | 943(83%) | 236(84%) | 255(84%) | 228(80%) | | 224(83%) |  | |
| Cancer | | 1,167 |  |  |  |  | |  | 0.7 | |
| yes | |  | 250(23%) | 67(24%) | 64(22%) | 51(20%) | | 68(26%) |  | |
| no | |  | 917(77%) | 224(76%) | 251(78%) | 234(80%) | | 208(74%) |  | |

All estimates were adjusted for the survey weights of NHANES（Q1：N = 1,389,3792,Q2：N = 1,386,9012,Q3：N = 1,384,8842,Q4：N = 1,386,6342）. Baseline characteristics of participants. Continuous variables are presented as the weighted mean ± standard error. Categorical variables are presented as an unweighted number (%). All estimates were adjusted for the survey weights of NHANES. NHR:neutrophil to high-density lipoprotein cholesterol ratio; BMI: body mass index; PIR: the poverty income ratio ;HDL-C: high-density lipoprotein cholesterol; TC: total cholesterol; TG: triglycerides; WBC: white blood cell count; PLT: platelet count; AST: aspartate aminotransferase; ALT: alanine aminotransferase; FBG: glucose, refrigerated serum; HbA1c: glycosylated hemoglobin; WC: waist circumference; SBP: mean systolic blood pressure; DBP: mean diastolic blood pressure; CAD: Coronary Artery Disease; AP: Angina Pectoris; MI: Myocardial Infarction.
