# Supplementary material for: The uric acid-to-high-density lipoprotein cholesterol ratio, neutrophil-to-high-density lipoprotein cholesterol ratio, and lymphocyte-to-high-density lipoprotein cholesterol ratio as risk indicators for mortality in congestive heart failure: A cross-sectional analysis of NHANES 2003 to 2016
Source: Medicine (Baltimore). 2026 Jun 26;105(26):e49313. doi: 10.1097/MD.0000000000049313 (PMC13313641; doi:10.1097/MD.0000000000049313)
Supplement: Supplementary file 2 [file medi-105-e49313-s002.docx]

**Table S3 Univariate Cox regression analysis**

| **Variables** | **All-cause mortality  HR (95%CI)** | **P-value** | **CVD mortality  HR (95%CI)** | **P-value** |
| --- | --- | --- | --- | --- |
| **UHR** | 1.03(1.01, 1.04) | 0.002 | 1.03(1.01, 1.05) | 0.001 |
| **UHR group** |  | 0.072 |  | 0.087 |
| Q1 | Reference |  | Reference |  |
| Q2 | 0.84(0.60, 1.16) |  | 1.07(0.68, 1.68) |  |
| Q3 | 1.00(0.74, 1.35) |  | 1.22(0.74, 2.03) |  |
| Q4 | 1.28(0.94, 1.72) |  | 1.59(1.08, 2.35) |  |
| **SUA** | 1.17(1.11, 1.24) | <0.001 | 1.20(1.11, 1.30) | <0.001 |
| **HDL-C** | 1.04(0.79, 1.36) | 0.784 | 1.00(0.99, 1.01) | 0.989 |
| **NHR** | 14.1(2.60, 76.6) | 0.002 | 34.7(2.92, 413) | 0.005 |
| **NHR group** |  | 0.012 |  | 0.013 |
| Q1 | Reference |  | Reference |  |
| Q2 | 1.15(0.87, 1.54) |  | 1.12(0.68, 1.87) |  |
| Q3 | 1.30(0.92, 1.83) |  | 1.52(0.95, 2.43) |  |
| Q4 | 1.57(1.18, 2.10) |  | 1.78(1.16, 2.73) |  |
| **LHR** | 0.32(0.19, 0.54) | <0.001 | 0.31(0.14, 0.71) | 0.005 |
| **LHR group** |  | <0.001 |  | 0.018 |
| Q1 | Reference |  | Reference |  |
| Q2 | 0.68(0.50, 0.91) |  | 0.66(0.45, 0.98) |  |
| Q3 | 0.65(0.49, 0.88) |  | 0.56(0.35, 0.89) |  |
| Q4 | 0.44(0.32, 0.61) |  | 0.50(0.32, 0.78) |  |
| **Gender** |  | 0.484 |  | 0.515 |
| male | Reference |  | Reference |  |
| female | 1.10(0.84, 1.44) |  | 0.88(0.61, 1.28) |  |
| **Age** | 1.19(0.98, 1.45) | 0.084 | 1.25(0.95, 1.64) | 0.114 |
| **AGE group** |  | <0.001 |  | <0.001 |
| 21-64 | Reference |  | Reference |  |
| ≥65 | 2.78(2.23, 3.45) |  | 3.38(2.21, 5.14) |  |
| **Race** |  | 0.003 |  | 0.298 |
| Mexican | Reference |  | Reference |  |
| American | 0.46(0.24, 0.89) |  | 0.7(0.27, 1.85) |  |
| Other Hispanic | 1.12(0.79, 1.58) |  | 1.32(0.69, 2.52) |  |
| Non-Hispanic | 0.74(0.48, 1.12) |  | 1.13(0.54, 2.38) |  |
| White | 0.97(0.56, 1.69) |  | 1.06(0.36, 3.15) |  |
| **Eduction** |  | 0.004 |  | 0.620 |
| Less Than 9th Grade | Reference |  | Reference |  |
| 9-11th Grade | 0.72(0.54, 0.98) |  | 0.87(0.57, 1.33) |  |
| High School Grad/GED | 0.72(0.49, 1.05) |  | 0.82(0.48, 1.39) |  |
| Some College | 0.58(0.43, 0.79) |  | 0.74(0.46, 1.18) |  |
| College Graduate or above | 0.58(0.40, 0.84) |  | 0.69(0.40, 1.19) |  |
| **Marital** |  | <0.001 |  | <0.001 |
| Married | Reference |  | Reference |  |
| Widowed | 2.28(1.77, 2.95) |  | 2.61(1.85, 3.67) |  |
| Divorced | 1.18(0.88, 1.59) |  | 1.19(0.73, 1.92) |  |
| Separated | 0.65(0.30, 1.45) |  | 0.36(0.08, 1.61) |  |
| Single | 0.57(0.31, 1.05) |  | 0.76(0.31, 1.89) |  |
| Living with partner | 0.50(0.22, 1.13) |  | 0.53(0.18, 1.58) |  |
| **PIR** | 0.9(0.83, 0.97) | 0.007 | 0.87(0.77, 0.98) | 0.028 |
| **PIR group** |  | 0.002 |  | 0.028 |
| <1.30 | Reference |  | Reference |  |
| 1.31-3.50 | 1.02(0.82, 1.27) |  | 1.1(0.83, 1.45) |  |
| ≥3.50 | 0.64(0.47, 0.87) |  | 0.56(0.33, 0.97) |  |
| **SBP** | 1.01(1.00, 1.01) | <0.001 | 1.01(1.01, 1.02) | <0.001 |
| **DBP** | 0.98(0.98, 0.99) | <0.001 | 0.99(0.98, 1.00) | 0.082 |
| **BMI** | 0.98(0.97, 1.00) | 0.018 | 0.98(0.96, 1.00) | 0.022 |
| **BMI group** |  | 0.170 |  | 0.213 |
| <25 | Reference |  | Reference |  |
| 25-30 | 0.8(0.60, 1.08) |  | 0.75(0.48, 1.17) |  |
| ≥30 | 0.76(0.57, 1.01) |  | 0.71(0.49, 1.04) |  |
| **WAIST** | 1.00(0.99, 1.00) | 0.146 | 0.99(0.98, 1.00) | 0.069 |
| **SCr** | 1.00(1.00, 1.00) | 0.001 | 1.00(1.00, 1.00) | 0.110 |
| **TG** | 0.97(0.91, 1.03) | 0.306 | 1.00(1.00, 1.00) | 0.918 |
| **LDL-C** | 1.00(0.96, 1.04) | 0.976 | 1.00(1.00, 1.00) | 0.954 |
| **TC** | 1.00(1.00, 1.00) | 0.058 | 1.00(1.00, 1.00) | 0.356 |
| **WBC** | 1.06(1.02, 1.10) | 0.002 | 1.06(1.00, 1.12) | 0.059 |
| **Lymphocyte** | 0.96(0.94, 0.97) | <0.001 | 0.95(0.93, 0.97) | 0.001 |
| **Monocyte** | 1.05(1.01, 1.08) | 0.004 | 1.07(1.03, 1.11) | <0.001 |
| **Neutrophil** | 1.12(1.06, 1.18) | <0.001 | 1.13(1.05, 1.23) | 0.002 |
| **PLT** | 1.00(1.00, 1.00) | 0.547 | 1.00(1.00, 1.00) | 0.134 |
| **ALB** | 0.37(0.28, 0.48) | 0.119 | 1.01(1.00, 1.01) | 0.002 |
| **HbA1c** | 1.12(1.04, 1.21) | 0.002 | 1.17(1.04, 1.31) | 0.008 |
| **FBG** | 1.00(1.00, 1.00) | 0.032 | 1.00(1.00, 1.01) | 0.036 |
| **ALT** | 1.00(0.99, 1.01) | 0.969 | 0.98 (0.96, 1.00) | 0.039 |
| **AST** | 1.01(1.00, 1.01) | 0.004 | 1.00(0.99, 1.01) | 0.765 |
| **ALP** | 1.01(1.00, 1.01) | <0.001 | 1.00(1.00, 1.01) | 0.071 |
| **BUN** | 1.04(1.03, 1.05) | <0.001 | 1.04(1.03, 1.05) | <0.001 |
| **LDH** | 1.01(1.00, 1.01) | <0.001 | 1.01(1.00, 1.01) | 0.002 |
| **P** | 1.40(1.19, 1.65) | <0.001 | 1.69(1.39, 2.07) | <0.001 |
| **TBil** | 1.44(1.00, 2.07) | 0.051 | 1.92(1.23, 2.99) | 0.004 |
| **HT** |  | 0.150 |  | 0.116 |
| no | Reference |  | Reference |  |
| yes | 1.20(0.94, 1.55) |  | 1.4(0.92, 2.14) |  |
| **DM** |  | <0.001 |  | 0.002 |
| no | Reference |  | Reference |  |
| yes | 1.50(1.28, 1.75) |  | 1.63(1.19, 2.23) |  |
| **Smoke** |  | 0.001 |  | 0.113 |
| Never | Reference |  | Reference |  |
| Former | 0.79(0.58, 1.07) |  | 0.58(0.35, 0.97) |  |
| Current | 1.32(1.07, 1.63) |  | 0.89(0.65, 1.21) |  |
| **Alcho** |  | 0.110 |  | 0.151 |
| Never | Reference |  | Reference |  |
| Former | 0.83(0.65, 1.04) |  | 0.72(0.51, 1.03) |  |
| Current | 0.76(0.57, 1.01) |  | 0.71(0.44, 1.14) |  |
| **CAD** |  | 0.004 |  | 0.035 |
| yes | Reference |  | Reference |  |
| no | 0.70(0.55, 0.89) |  | 0.69(0.49, 0.97) |  |
| **AP** |  | 0.722 |  | 0.505 |
| yes | Reference |  | Reference |  |
| no | 1.04(0.83, 1.31) |  | 1.12(0.80, 1.57) |  |
| **MI** |  | 0.949 |  | 0.969 |
| yes | Reference |  | Reference |  |
| no | 0.99(0.83, 1.19) |  | 1.01(0.76, 1.33) |  |
| **Stroke** |  | <0.001 |  | <0.001 |
| yes | Reference |  | Reference | 0.008 |
| no | 0.61(0.48, 0.77) |  | 0.61(0.48, 0.77) |  |
| **cancer** |  | 0.002 |  | 0.050 |
| yes | Reference |  | Reference |  |
| no | 0.68(0.53, 0.87) |  | 0.71(0.51, 1.00) |  |
