# Supplementary figures and images for: The uric acid-to-high-density lipoprotein cholesterol ratio, neutrophil-to-high-density lipoprotein cholesterol ratio, and lymphocyte-to-high-density lipoprotein cholesterol ratio as risk indicators for mortality in congestive heart failure: A cross-sectional analysis of NHANES 2003 to 2016
Source: Medicine (Baltimore). 2026 Jun 26;105(26):e49313. doi: 10.1097/MD.0000000000049313 (PMC13313641; doi:10.1097/MD.0000000000049313)

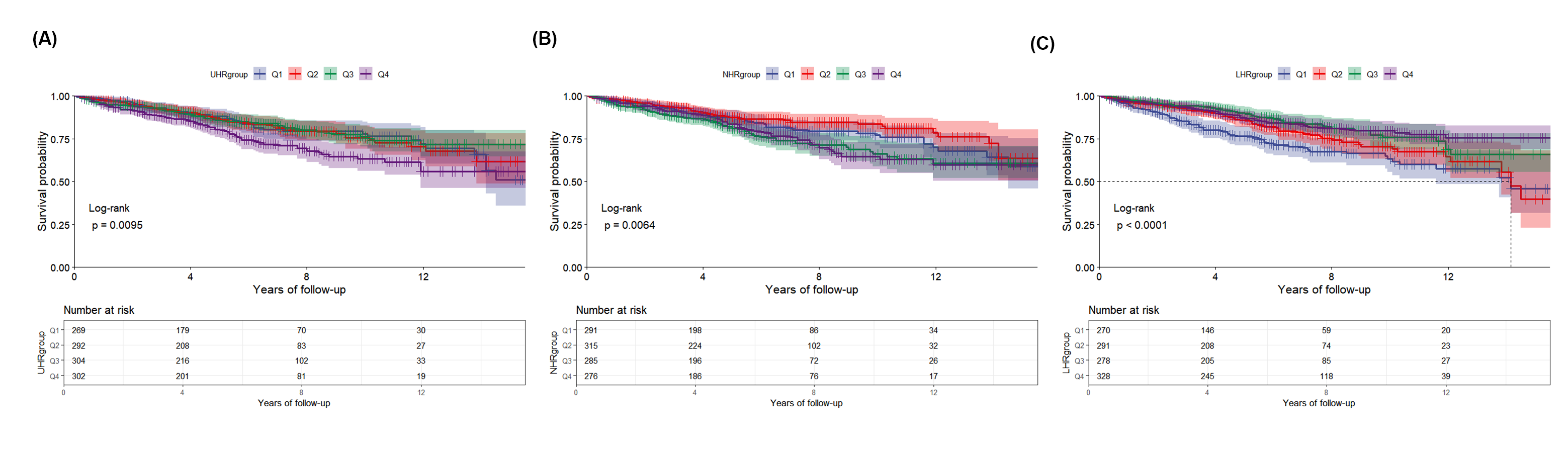

Supplement: Supplementary file 3 [file medi-105-e49313-s003.tiff]

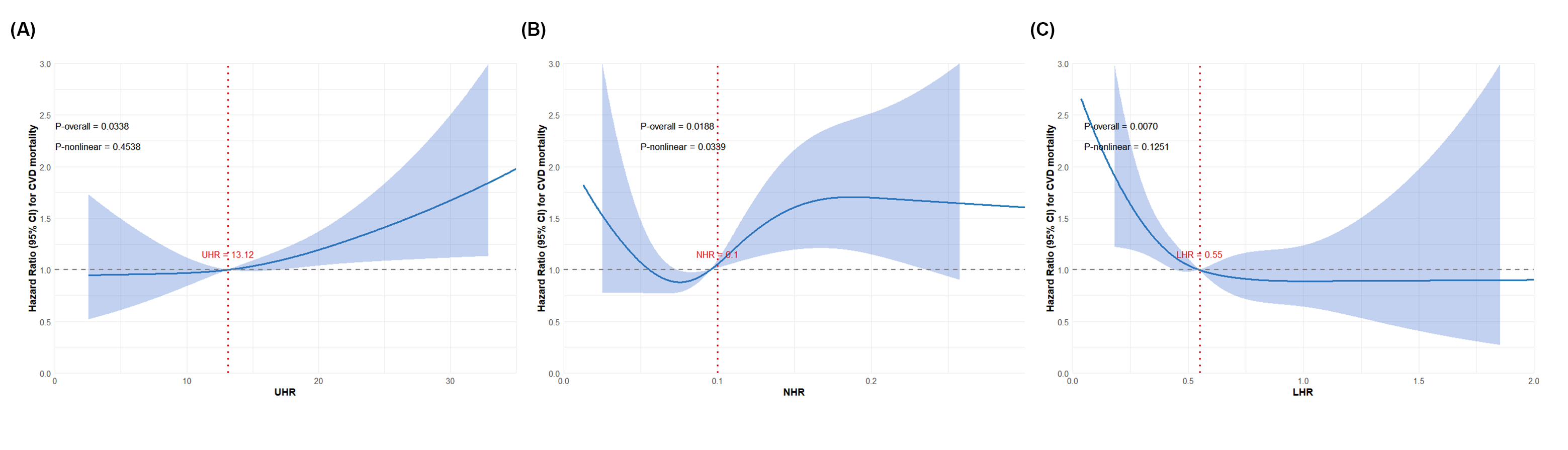

Supplement: Supplementary file 4 [file medi-105-e49313-s004.tiff]

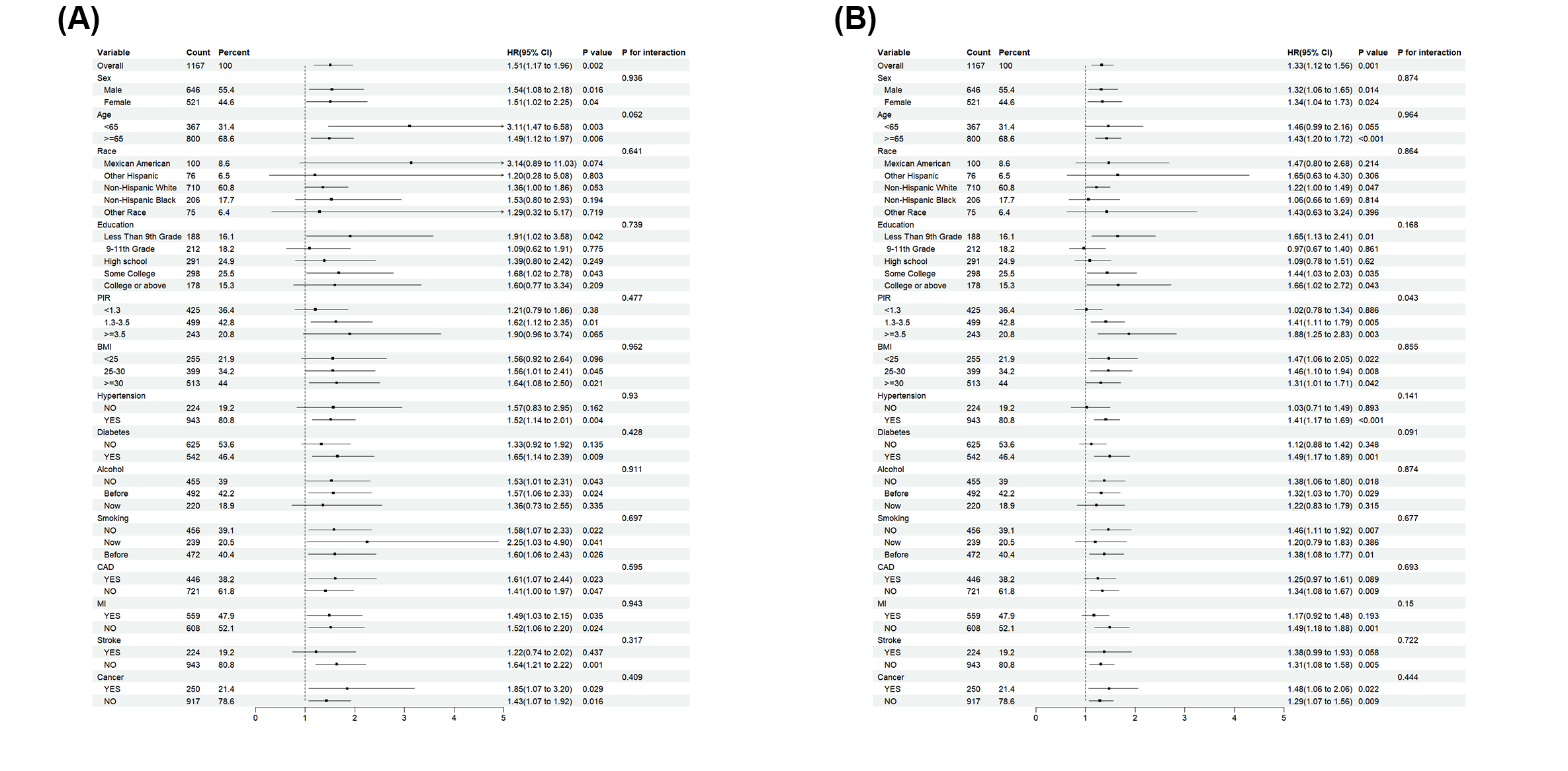

Supplement: Supplementary file 5 [file medi-105-e49313-s005.tiff]

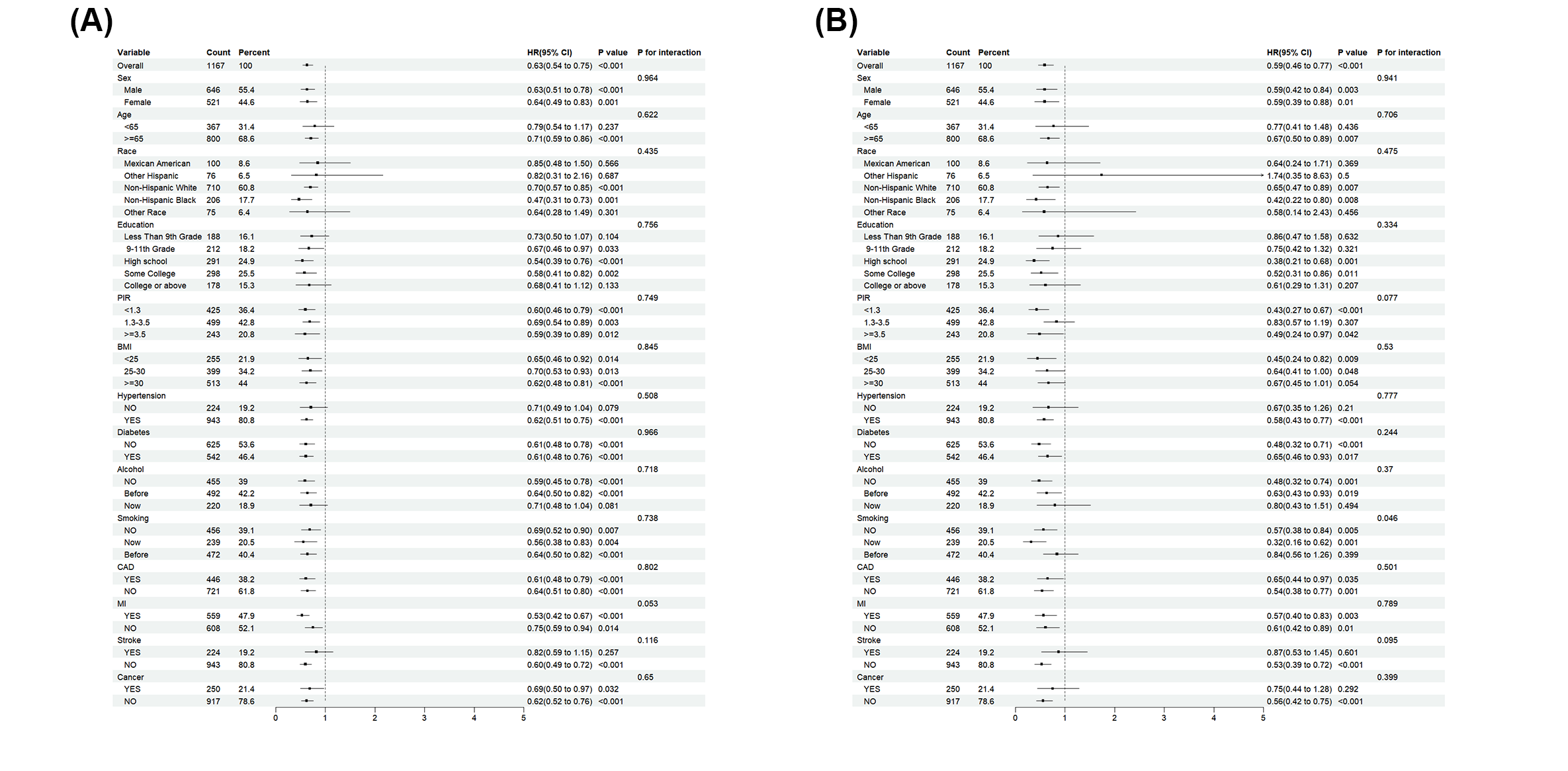

Supplement: Supplementary file 6 [file medi-105-e49313-s006.tiff]

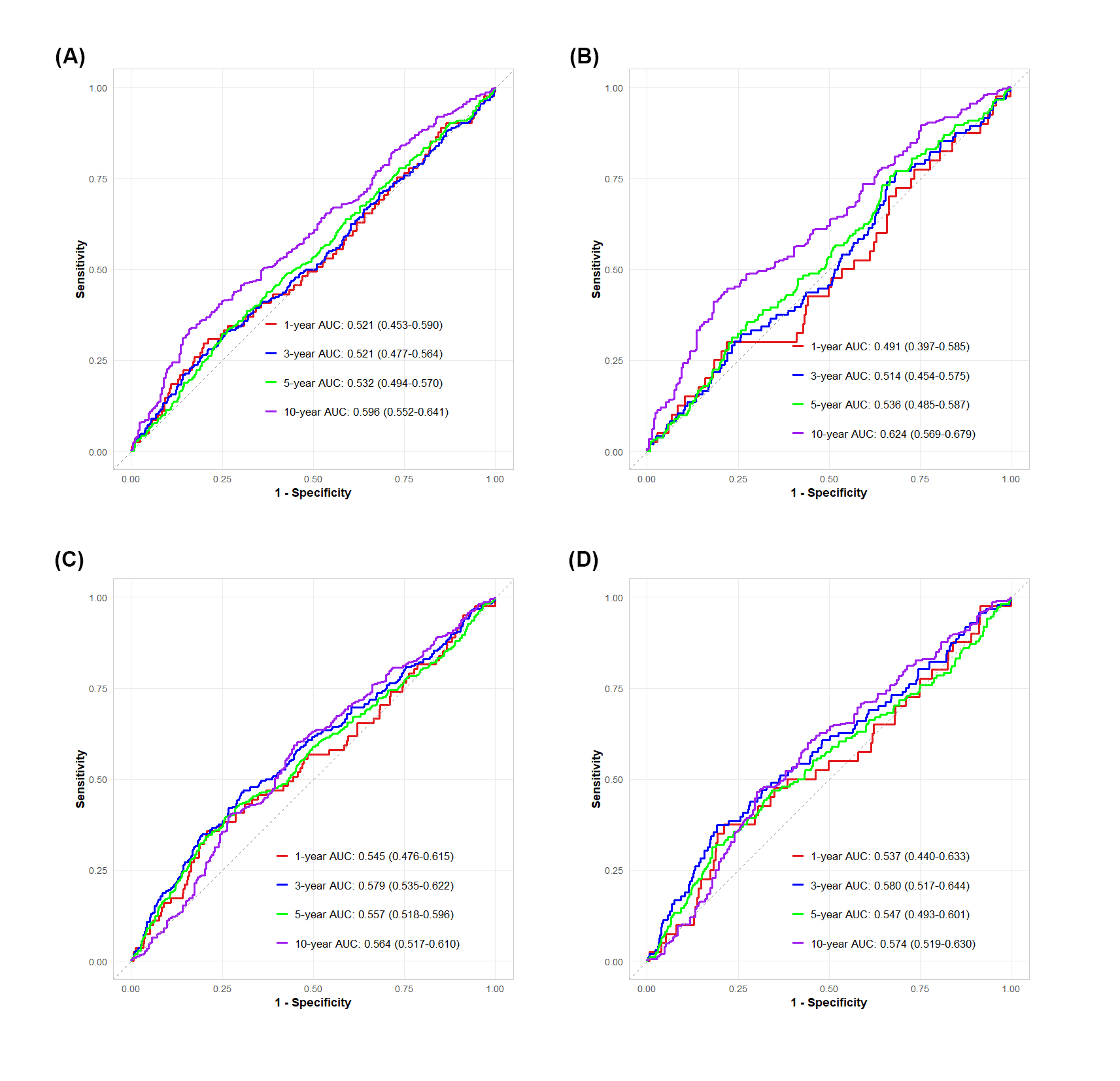

Supplement: Supplementary file 7 [file medi-105-e49313-s007.tiff]

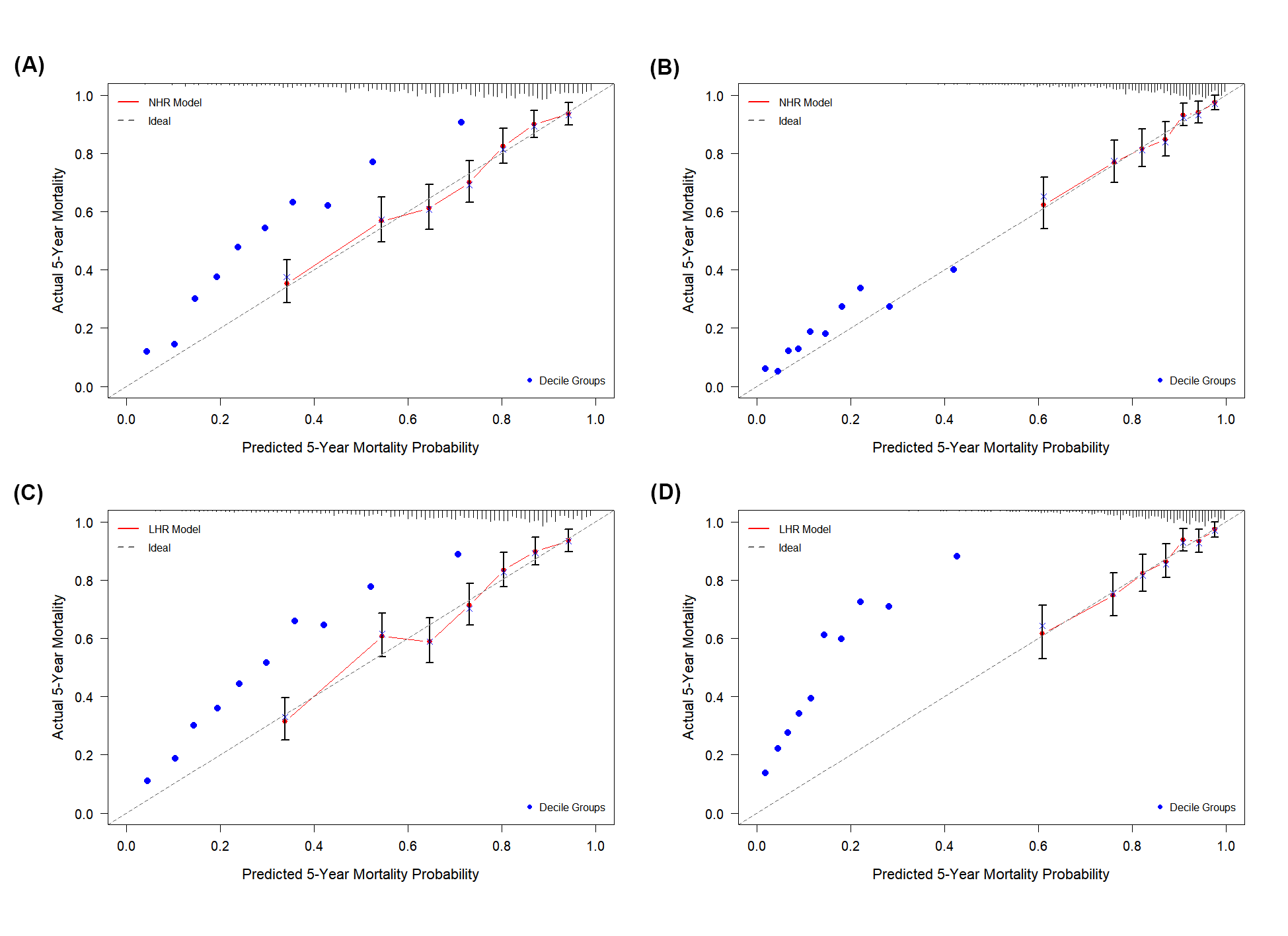

Supplement: Supplementary file 9 [file medi-105-e49313-s009.tiff]

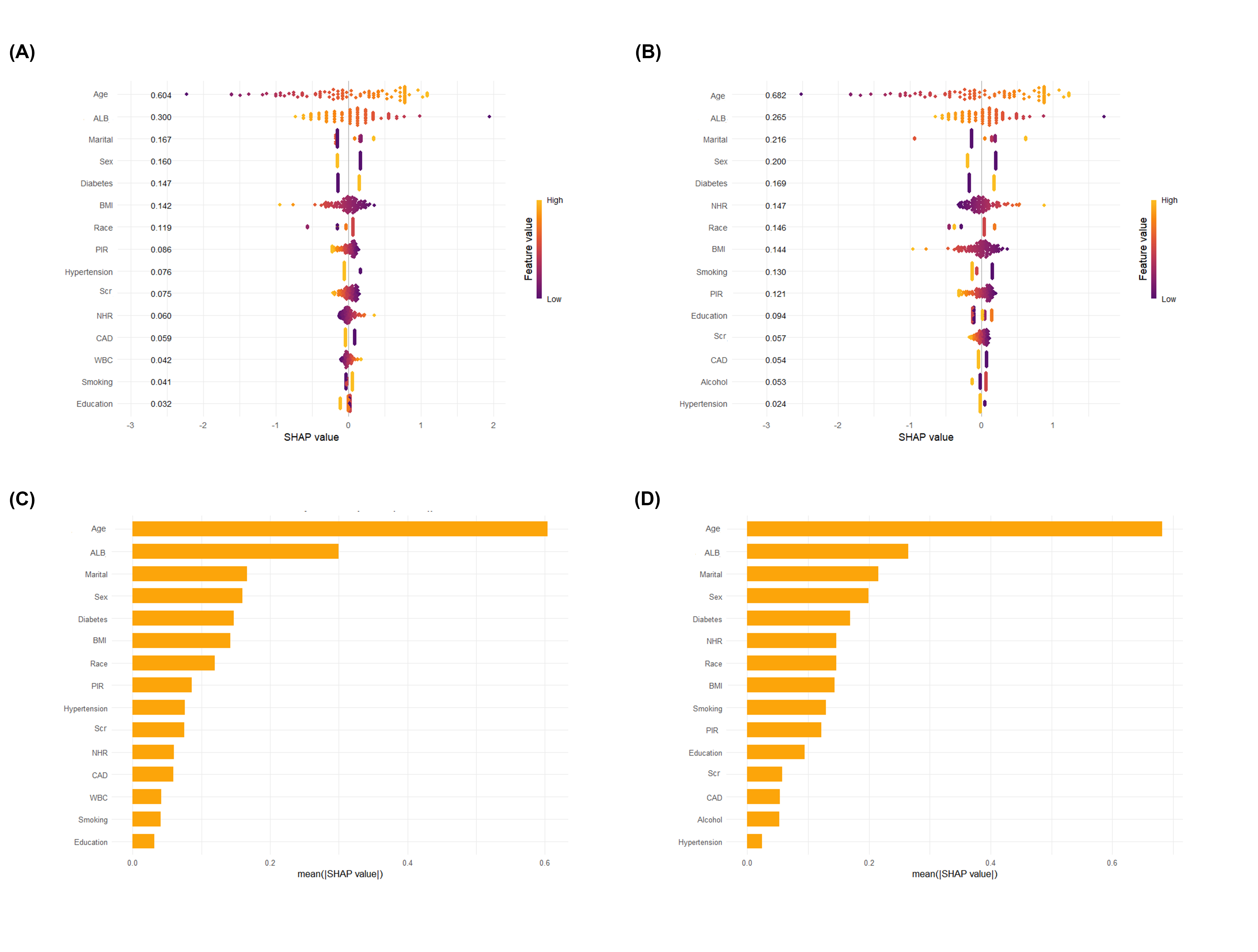

Supplement: Supplementary file 10 [file medi-105-e49313-s010.tif]

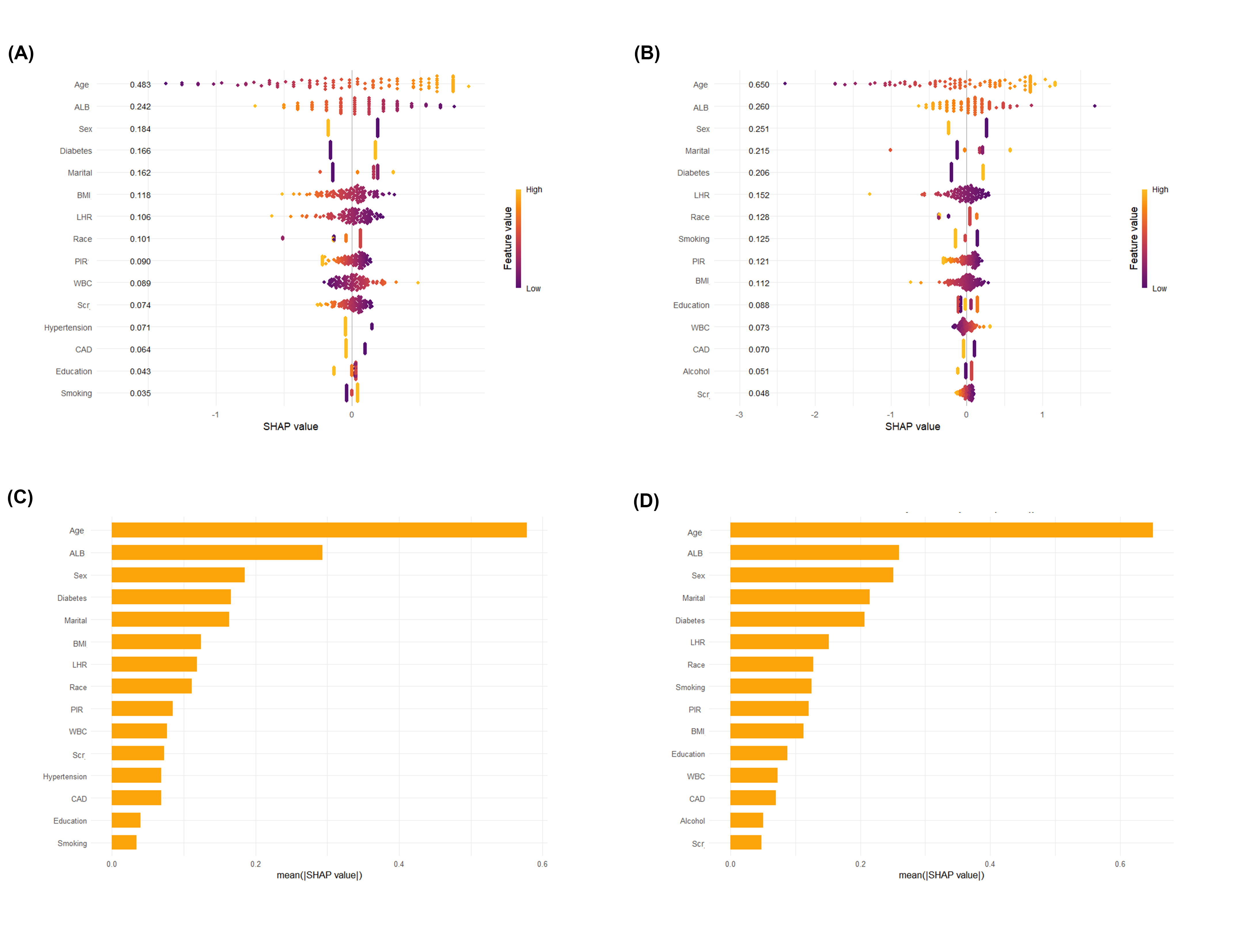

Supplement: Supplementary file 12 [file medi-105-e49313-s012.tif]
