# Supplementary material for: The uric acid-to-high-density lipoprotein cholesterol ratio, neutrophil-to-high-density lipoprotein cholesterol ratio, and lymphocyte-to-high-density lipoprotein cholesterol ratio as risk indicators for mortality in congestive heart failure: A cross-sectional analysis of NHANES 2003 to 2016
Source: Medicine (Baltimore). 2026 Jun 26;105(26):e49313. doi: 10.1097/MD.0000000000049313 (PMC13313641; doi:10.1097/MD.0000000000049313)
Supplement: Supplementary file 8 [file medi-105-e49313-s008.docx]

****Table S4** Summary of areas under the curve and 95% confidence interval for receiver operating characteristic Curves**

**Table S4.1** Area Under the Curve and 95% Confidence Intervals for uric acid to high-density lipoprotein cholesterol ratio, neutrophil to high-density lipoprotein cholesterol ratio, and lymphocyte to high-density lipoprotein cholesterol ratio in Predicting All-Cause and Cardiovascular Mortality

|  | **all-cause mortality** | | **cardiovascular mortality** | |
| --- | --- | --- | --- | --- |
| **ROC curves** | AUC values | 95%CI | AUC values | 95%CI |
| UHR | 0.55 | 0.51-0.60 | 0.56 | 0.51-0.61 |
| NHR | 0.55 | 0.51-0.60 | 0.56 | 0.51-0.61 |
| LHR | 0.59 | 0.55-0.64 | 0.57 | 0.51-0.61 |

**Table S4.2** Time-Dependent Area Under the Curve of uric acid to high-density lipoprotein cholesterol ratio, neutrophil to high-density lipoprotein cholesterol ratio, and lymphocyte to high-density lipoprotein cholesterol ratio for Predicting All-Cause and Cardiovascular Mortality at 1, 3, 5, and 10 Years

|  | **time-dependent AUC values** | 1year | 3year | 5year | 10year |
| --- | --- | --- | --- | --- | --- |
| **all-cause mortality** | UHR | 0.581 | 0.541 | 0.538 | 0.563 |
|  | LHR | 0.545 | 0.579 | 0.557 | 0.564 |
|  | NHR | 0.521 | 0.521 | 0.532 | 0.596 |
| **cardiovascular mortality** | UHR | 0.610 | 0.571 | 0.556 | 0.586 |
|  | LHR | 0.537 | 0.580 | 0.547 | 0.574 |
|  | NHR | 0.491 | 0.514 | 0.536 | 0.624 |

Adjusting for gender, age, race/ethnicity, education, marital status, PIR, BMI, SCr, WBC, ALB, TC, diabetes, hypertension, CAD, smoking status, and drinking status. ROC: receiver operating characteristic. AUC: areas under the curve. UHR: uric acid and high-density lipoprotein cholesterol ratio. NHR: the neutrophil-to-HDL cholesterol ratio. LHR:lymphocyte-to-HDL cholesterol ratio .
